# Acute transient cognitive dysfunction and acute brain injury induced by systemic inflammation occur by dissociable IL-1-dependent mechanisms

**DOI:** 10.1038/s41380-018-0075-8

**Published:** 2018-06-06

**Authors:** Donal T. Skelly, Éadaoin W. Griffin, Carol L. Murray, Sarah Harney, Conor O’Boyle, Edel Hennessy, Marc-Andre Dansereau, Arshed Nazmi, Lucas Tortorelli, J. Nicholas Rawlins, David M. Bannerman, Colm Cunningham

**Affiliations:** 1 School of Biochemistry and Immunology & Trinity College Institute of Neuroscience, Dublin 2, Ireland; 2 Department of Physiology, Trinity College Dublin, Dublin 2, Ireland; 3 Department of Experimental Psychology, University of Oxford, South Parks Road, Oxford, UK

**Keywords:** Neuroscience, Physiology, Psychology

## Abstract

Systemic inflammation can impair cognition with relevance to dementia, delirium and post-operative cognitive dysfunction. Episodes of delirium also contribute to rates of long-term cognitive decline, implying that these acute events induce injury. Whether systemic inflammation-induced acute dysfunction and acute brain injury occur by overlapping or discrete mechanisms remains unexplored. Here we show that systemic inflammation, induced by bacterial LPS, produces both working-memory deficits and acute brain injury in the degenerating brain and that these occur by dissociable IL-1-dependent processes. In normal C57BL/6 mice, LPS (100 µg/kg) did not affect working memory but impaired long-term memory consolidation. However prior hippocampal synaptic loss left mice selectively vulnerable to LPS-induced working memory deficits. Systemically administered IL-1 receptor antagonist (IL-1RA) was protective against, and systemic IL-1β replicated, these working memory deficits. Dexamethasone abolished systemic cytokine synthesis and was protective against working memory deficits, without blocking brain IL-1β synthesis. Direct application of IL-1β to ex vivo hippocampal slices induced non-synaptic depolarisation and irreversible loss of membrane potential in CA1 neurons from diseased animals and systemic LPS increased apoptosis in the degenerating brain, in an IL-1RI-dependent fashion. The data suggest that LPS induces working memory dysfunction via circulating IL-1β but direct hippocampal action of IL-1β causes neuronal dysfunction and may drive neuronal death. The data suggest that acute systemic inflammation produces both reversible cognitive deficits, resembling delirium, and acute brain injury contributing to long-term cognitive impairment but that these events are mechanistically dissociable. These data have significant implications for management of cognitive dysfunction during acute illness.

## Introduction

Peripheral infections are known to trigger episodes of acute cognitive impairment, including delirium, in older populations and in those with dementia [1, 2]. Sterile inflammation, resulting from tissue trauma or surgery, can also induce post-operative cognitive dysfunction and delirium [3, 4]. Cytokines are key mediators of septic and aseptic inflammation and, given its important role in coordinating CNS responses to systemic inflammation [5, 6], the pro-inflammatory cytokine IL-1β might be predicted equally to underlie infection- and sterile inflammation-induced cognitive dysfunction. Consistent with this idea, IL-1β levels have been associated with delirium in hip fracture patients and in septic encephalopathy [7, 8]. Delirium is a profound and acute onset brain dysfunction with impairments in attention and other aspects of cognition. Its high prevalence after surgery and infection emphasises the deleterious consequences that systemic inflammation has for cognitive function, particularly in older, cognitively impaired, populations [9, 10]. It is now clear that acute systemic inflammation and delirium also significantly increase the risk of long-term cognitive decline and dementia [11–13], and accelerate the course of existing dementia [14, 15]. Despite the enormous medical and economic implications [16] of these findings, whether systemic inflammation-induced acute dysfunction and acute brain injury occur by overlapping or discrete mechanisms has not been invest

It is established that IL-1 impacts on cognitive function [17, 18]. IL-1 disrupts consolidation of context-associated fear memory [19–21] and IL-1 receptor antagonist (IL-1RA) is protective against post-acquisition memory consolidation deficits induced by both surgery and systemic lipopolysaccharide (LPS) [20, 22, 23]. However, it is likely that systemic inflammation impacts upon multiple cognitive processes. Furthermore, long-term memory consolidation is manifestly different both from the acute fluctuating short-term memory processes that are affected in delirium and in relevant models [9, 24] and from acute neuronal death events described after systemic inflammation [25]. Given the massive public health burden of dementia and delirium, both of which have been associated with IL-1 [26,27], it is important to characterise differential roles of IL-1β in these processes. In the current study we hypothesised that (1) bacterial endotoxin (LPS; lipopolysaccharide) would have multiple effects on cognition and neuronal integrity and that (2) IL-1 would be causative in these changes.

## Materials and methods

### Animals

Female C57BL/6 mice at 8–12 weeks of age (Harlan Olac Ltd, UK) were housed in cages of 5 at 21 °C with a 12:12 h light–dark cycle with food and water ad libitum. To test the effects of LPS in cognitive function in young healthy mice (2–4 months) C57BL/6 mice were injected intraperitoneally (i.p.) with 100 (or 200) μg/kg of LPS (*Salmonella Equine abortus*, Sigma (L5886)) in sterile saline. To examine effects of LPS in animals with prior synaptic loss mice were first intrahippocampally inoculated with 1 µl of 10% w/v prion infected- (ME7) or normal brain homogenate (NBH), at mm from bregma: anterior–posterior −2.0, lateral −1.7, depth −1.6) using a Hamilton microsyringe, under anaesthesia with intra-peritoneal 2,2,2-tribromoethanol. Experimental groups (ME7, NBH) were then injected i.p. with 100 μg/kg of LPS at 15–16 weeks post inoculation (demonstrated vulnerability to LPS-induced working-memory deficits [28]). Control animals received non-pyrogenic saline.

Alternative inflammatory stimuli and anti-inflammatory interventions were also examined: IL-1β (R&D Systems, Minneapolis, MN, USA, 401-ML/CF) and TNF-α (Peprotech, Rocky Hill, NJ, USA, 305-01A) were injected at 15 µg/kg at 50 µg/kg respectively in sterile saline (i.p.). IL-1RA (10 mg/kg, i.p.; Kineret, Biovitrum, Sweden) was given immediately before LPS or IL-1β (15 µg/kg); dexamethasone-21-phosphate (Sigma, D1159) was administered i.p. at a dose of 2 mg/kg, 60 min before LPS (sufficient to robustly suppress the systemic secretion of IL-1β, TNF-α and IL-6 [29]. Samples sizes for all groups in all animal experiments were chosen on the basis of our prior published studies using the ME7 + LPS paradigm [25, 28, 30] and are specified in the appropriate figure legends.

IL-1RI^−/−^ mice (B6.129S7-Il1r1<tm1Imx>; kindly provided by Prof. Kingston Mills) have a null mutation in *Il1r1* and were backcrossed seven times to C57BL/6 before maintenance as an inbred colony (with C57BL/6 as controls). IL-1RI^−/−^ and C57BL/6 mice were not significant different in working memory or contextual fear conditioning (CFC) tasks [31]. Body temperature was measured using a rectal probe (TH-5 thermoprobe, Physitemp, NJ) 18 h post challenge with LPS (750 µg/kg i.p.) in WT and IL-1RI^−/−^ mice inoculated with ME7 or NBH.

All animal procedures were performed in accordance with Irish Department of Health & Children, Health Products Regulatory Authority and UK Home Office regulations and all efforts were made to minimise suffering to the animals.

### Working memory: Food-rewarded and escape from water T-maze alternation tasks

We assessed short-term/working memory using alternation behaviour in both food-rewarded and escape-from-water-motivated T-maze tasks (3–9 h post LPS and 1 to 7 h post IL-1β). These times were based on pilot data and are justified by the recovery by 5 h in IL-1-treated mice. In both cases mice were placed in the start arm of the maze with one arm of the T blocked such that they were forced to make a left (or right) turn, predetermined by a pseudo-random sequence (equal numbers of left and right turns, no more than two consecutive runs to the same arm). Mice could then escape the maze via an escape tube before being held in a holding cage for 25 s (water version) or could remain in the forced arm for 30 s to consume 70 µl of sweetened condensed milk (food-rewarded version). Mice were then replaced in the start arm and had to choose the alternate arm to once again escape the maze or to receive a second food reward. Ten or 15 trials were performed on these tasks under the influence of systemic inflammation. Only in animals who had achieved a criterion baseline performance of ≥70% (water) or ≥80% (food-rewarded) alternation for 2 or more consecutive days. Baseline T-maze performance was ranked and animals were assigned to different treatments groups such that all treatment groups had equivalent, or very similar, baseline performance. Experimenters were blind to treatment during scoring of cognitive function. This water-escape task has previously been published [28] and a full description of these methods is available in supplementary material.

### Contextual fear conditioning

CFC was recorded using a clear perspex box (40 cm × 10 cm × 16 cm) with a floor containing metal rods connected to a shock generator (UGO Basile, Italy). The mice were placed into the box and allowed to explore for 2 min. A tone at 2.9 kHz for 20 s was presented, followed by a shock of 0.4 mA for 2 s. This tone/shock pairing was repeated after 2 min. After a further 30 s of exploration mice were removed to a holding cage. After 30 s in the holding cage saline or LPS (±saline or IL-1RA, at discrete i.p. sites) were administered before returning the animals to the home cage. IL-1RA was administered at this time (rather than preceding the test of retention) because it is consolidation, rather than retention, of memory that has been shown to be impaired by LPS [32, 33]. Fear conditioning was assessed for 5 min in the same location 48 h later. Freezing was defined as the complete absence of movement, except those related to respiration [34]. Auditory fear conditioning was also assessed 48 h post-fear conditioning. Animals were placed in a different context (a novel empty cage) for 6 min and were allowed to explore for 3 min (baseline) before presentation of the tone for 20 s. The time spent freezing during the final 3 min was recorded (i.e. during and post tone).

### ELISA for cytokines

Under terminal anaesthesia, blood was collected directly from the right atrium into heparinised tubes, was centrifuged at 3000 rpm for 15 min at 4 °C and the remaining plasma aliquoted and stored at −20 °C. Samples were then analysed for CCL2 and CXCL1 using R&D systems sandwich-type duo set ELISA kits (DY479, DY453) while IL-1β was analysed using a Quantikine kit (R&D systems, Minneapolis, MN, USA, MLB00C). A standard protocol was followed as previously described [30] except for IL-1β, which was as per manufacturer’s instructions with minor modifications. To ensure that all cytokines were reliably quantifiable using the appropriate standard curves, samples were serially diluted for CCL2 and CXCL1 (1/9, 1/81 and 1/243). Blood and brain samples were also assayed for the presence of human IL-1RA using an R&D Systems quantikine assay (DRA00B), performed according to manufacturers’ instructions (standards 0–2000 pg/ml). Hippocampal/thalamic tissue punches were homogenised in 150 mM NaCl, 25 mM Tris-HCl and 1% Triton X100 at pH 7.4 before centrifugation at 14,000 rpm for 10 min. Supernatants were diluted 1 in 2 in assay diluent in wells pre-coated with anti-human IL-1RA polyclonal antibody.

### Quantitative PCR

The isolation of total RNA, synthesis of cDNA and analysis of transcription by quantitative PCR were performed as previously described [35]. Briefly, after transcardial perfusion, the hippocampus and dorsal thalamus were punched out of 2 mm thick coronal brain sections, snap frozen in liquid nitrogen and stored at −80 °C. Total RNA was extracted using Qiagen RNeasy Plus™ mini kits, with Qiashredders (Qiagen, Crawley, UK, #74134, #79654) according to manufacturer’s instructions. Contaminating gDNA was removed using the Qiagen RNase-free DNase I enzyme (Qiagen #79254). RNA yields were determined by spectrophotometry at 260 and 280 nm and stored at −80 °C. Using a High Capacity cDNA Reverse Transcriptase Kit (Applied Biosystems, Warrington, UK), cDNA was synthesised using 200 ng of total RNA in a 10 μl reaction volume. 1 μl of the reverse transcription reaction was used for quantitative PCR. Reagents were supplied by Applied Biosystems (SYBR® Green PCR Master Mix; 4364344) and Roche (FastStart Universal Probe Master [Rox], Lewes, UK; 04914058001). Assays were designed using Primer Express software and published sequences for the genes of interest. All primer sequences were as previously published [25]. Assays were quantified using a relative standard curve, as previously described [35] constructed from cDNA, synthesised from 1 μg total RNA isolated from mice showing up-regulation of all target transcripts of interest.

### Electrophysiology

Transverse hippocampal slices (300 mm) were prepared from brains of NBH or ME7 mice at 18–19 weeks post inoculation and from IL-1RI^−/−^ mice at 8 months of age. Slices were cut in ice-cold artificial cerebrospinal fluid (aCSF) solution containing (in mM) 75 sucrose, 87 NaCl, 25 NaHCO_3_, 2.5 KCl, 1.25 NaH_2_PO_4_, 0.5 CaCl_2_, 7 MgCl_2_, 10 d-glucose, 1 ascorbic acid and 3 pyruvic acid. During incubation and experiments slices were perfused with aCSF containing (in mM) 125 NaCl, 25 NaHCO_3_, 2.5 KCl, 1.25 NaH_2_PO_4,_ 2 CaCl_2_, 1 MgSO_4_ and 25 d-glucose. The slices were maintained at 33 °C for 1 h following dissection and all recordings were performed at physiological temperature (32–34 °C). Whole-cell patch-clamp recordings were made from CA1 pyramidal neurons, visualised using an upright microscope (Olympus BX51 WI, Middlesex, UK) with infra-red differential interference contrast optics (IR-DIC). Patch pipettes were filled with intracellular solution containing (in mM) 130 KMeSO_4_, 10 KCl, 0.2 EGTA, 10 HEPES, 20 phosphocreatine, 2 Mg_2_ATP, 0.3 NaGTP, 5 QX-314, 1 TEA (pH 7.3, 290–300 mOsm). Cells were voltage-clamped at −60 mV and input resistance and membrane capacitance were measured in response to a 10 mV depolarising voltage pulse. During current clamp recordings, current injection was initially set to maintain membrane potential at −60 mV and was not altered for the duration of experiments. Recordings were made using a Multiclamp 700B (Molecular Devices, Foster City, CA). Signals were filtered at 5 kHz using a 4-pole Bessel filter and were digitised at 10 kHz using a Digidata 1440 analogue-digital interface (Molecular Devices). Data were acquired and analysed using PClamp 10 and Clampfit (Molecular Devices). IL-1β was bath-applied to slices at concentrations of 0.1 ng/ml and 1 ng/ml.

### TUNEL immunohistochemistry

Immunohistochemistry for apoptotic cells was performed on formalin-perfused, wax embedded tissue from ME7 animals (wild-type and IL-1R1^−/−^), 18 h post LPS (750 µg/kg i.p.) using the ‘Dead End’ TUNEL staining method (Promega, Southampton, UK; G3250). Non-specific peroxidase activity was eliminated by incubating sections in 1% H_2_O_2_ for 10 min. Sections were washed in 0.85% NaCl for 5 min and PBS washed, before 5 min pre-treatment with proteinase K (10 μg/ml). After PBS washing, sections were preincubated with equilibration buffer (10 min) and then with TUNEL buffer (22 μl equilibration buffer, 2.5 μl nucleotide mix, 0.5 μl TdT enzyme per section) for 2 h at 37 °C, labelling apoptotic cells with fluorescein. After reaction termination with 2× sodium citrate (15 min) and PBS washing sections were blocked with 10% normal goat serum (30 min) and incubated with biotinylated anti-fluorescein antibody (5 μg/ml). Thereafter the Avidin–Biotin-Complex protocol was performed according to manufacturers instructions, using H_2_O_2_ as substrate and diaminobenzidine as chromagen. TUNEL-positive cells that also showed evidence of nuclear condensation were counted in 10 µm coronal sections of ME7 animals treated with LPS in wild-type and IL-1R1^−/−^ mice. At least two sections were counted, by an observer blind to the experimental conditions and averaged for each animal.

### Statistical analyses

Behavioural data were compared by repeated measures ANOVA with Bonferroni post hoc tests performed after significant main effects. Molecular, electrophysiology and TUNEL data were analysed by two-way ANOVA, while temperature data were analysed by three-way ANOVA, followed by Bonferroni post hoc tests for a priori selected pairwise comparisons. Sample sizes and variance were similar for all key comparisons.

## Results

### Differential effects of LPS on different hippocampal-dependent cognitive tasks

**Fig. 1:**
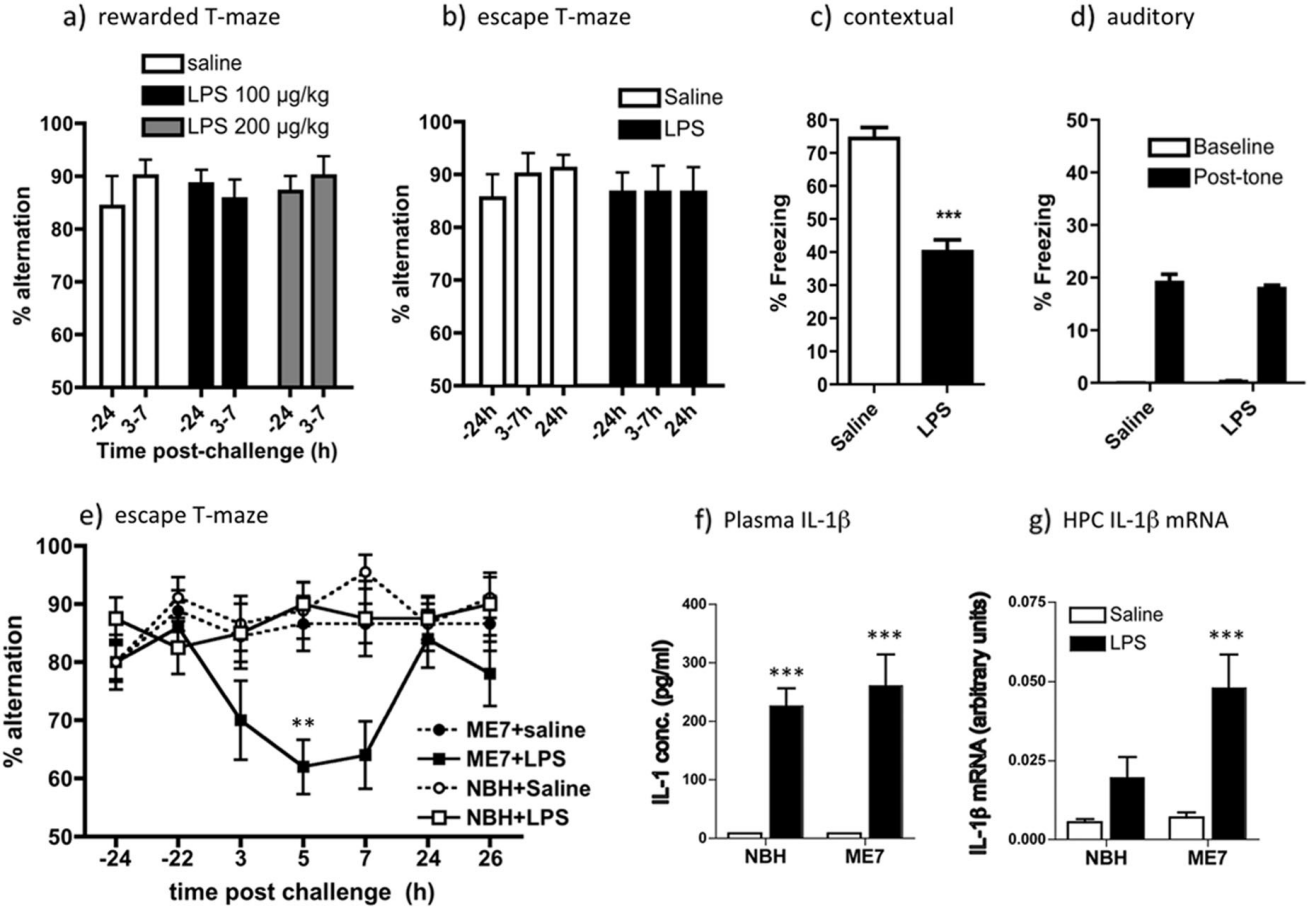
LPS-induced effects on working memory, fear conditioning and IL-1β expression. Working memory was assessed by (**a**) food-rewarded T-maze alternation (all groups *n* = 7) and (**b**) escape from shallow water T-maze (both groups *n* = 9) at 24 h before (−24) and at indicated times post LPS (100 or 200 μg/kg i.p.). On the *X*-axis, 3–7 represents % alternation during 10 trials conducted every 20 min between 3 and 7 h post LPS). Two-way ANOVA analysis found no significant effects of treatment. Contextual (**c**) and auditory (**d**) fear conditioning performance (freezing per 5 min) 48 h post challenge with LPS (100 μg/kg i.p.). Data are expressed as mean ± SEM and analysed by *t*-test (***p < 0.0001; *n* = 12 for saline and 16 for LPS; combined from two independent experiments). **e** Working memory performance of NBH and ME7 animals 16 weeks post-ME7 inoculation, challenged with LPS (100 μg/kg i.p.) or saline, assessed by T-maze alternation for 10 trials 24 h pre-challenge, 15 trials between 3–9 h post challenge and 10 trials 24 h post challenge. Full ANOVA analysis is described in the main text. Significant Bonferroni post hoc differences between ME7 + LPS and both NBH + LPS and ME7 + saline are denoted by ** (*p* < 0.01; ME7 + LPS *n* = 10, ME7 + saline, NBH + saline *n* = 9, NBH + LPS *n* = 8. **f** Plasma levels of IL-1β, were assessed by ELISA in 16-week ME7 and NBH animals 2 h post-LPS treatment (*n* = 4). Two-way ANOVA revealed a main effect of LPS challenge on circulating IL-1β (*F* = 60.06, df 1,12, *p* < 0.0001) but no main effect of disease and no interaction between treatment and disease (***p < 0.001, denotes the main effect of LPS, all groups *n* = 4). **g** Hippocampal transcription of IL-1β mRNA in 16-week ME7 and NBH animals, 2 h post-LPS treatment. Significant difference between ME7 + LPS and NBH + LPS groups (***p < 0.001 by Bonferroni post hoc after a significant interaction between disease and challenge by two-way ANOVA (*p* < 0.05; *n* = 7 for all groups except ME7 + LPS: *n* = 5). All data are presented as mean ± SEM

We assessed working memory using T-maze alternation tasks requiring both attention to, and retention in the working memory of (for 25 s), the prior location of an exit in order to determine the new exit location. LPS did not impair working memory in food-rewarded T-maze alternation at 3–7 h post treatment at either 100 or 200 µg/kg i.p. (Fig. 1a). To validate this finding in an aversively motivated working memory task, not reliant on motivation for appetitive rewards, we also used an ‘escape from shallow water’ T-maze working memory task specifically adapted for use in animals experiencing acute sickness behaviour. LPS (100 µg/kg) had no impact on working memory in C57BL6 mice in this T-maze (Fig. 1b).

However, LPS (100 µg/kg i.p.) given immediately post acquisition, was sufficient to significantly decrease freezing in CFC 48 h after exposure to context and foot-shock pairing (Fig. 1a, *p* < 0.001) but had no impact on auditory fear conditioning (Fig. 1d). In the CFC task LPS is known to impair consolidation (in the early hours after exposure to shock) rather than the retrieval of that memory 48 h later [32, 33]. Therefore, systemic LPS has differential effects on two hippocampal-dependent tasks: disrupting consolidation of contextual memory but not affecting working memory in normal animals.

### LPS differentially affects working memory in vulnerable animals despite equivalent circulating IL-1β

LPS (100 µg/kg) was sufficient to produce robust, acute and transient working memory deficits in animals with prior neurodegenerative pathology (ME7 model of prion disease). Impairment peaked at 5 h and had resolved by 24 h (Fig. 1e), replicating our prior studies [9]. LPS affected performance differently in NBH and ME7 animals (Repeated measures three-way ANOVA: interaction between disease and treatment, *F*_1,32_ = 4.64, *p* = 0.0388). ME7 + LPS at its nadir (5 h) was significantly different to both ME7 + saline and NBH + LPS (*p* < 0.01, Bonferroni post hoc). Therefore working memory remains intact when normal animals are LPS-challenged but is impaired when animals with neurodegeneration are similarly challenged. Despite the increased susceptibility of ME7 animals to working memory deficits in response to LPS, performance in the CFC task was equally susceptible to LPS disruption in normal and ME7 animals (Fig. S1).

This selective vulnerability of ME7 animals to LPS could potentially be underpinned by differential circulating IL-1β. Peripherally administered LPS (100 µg/kg) induced robust systemic IL-1β synthesis and IL-1β mRNA transcription in the hippocampus at 2 h post challenge. Plasma IL-1β levels were equivalent in ME7 and NBH animals (Fig. 1f), although brain IL-1β mRNA was exaggerated in ME7 + LPS (Fig. 1g), as previously described [25, 28].

### Dissociable effects of IL-1RA treatment on LPS-induced cognitive deficits

**Fig. 2:**
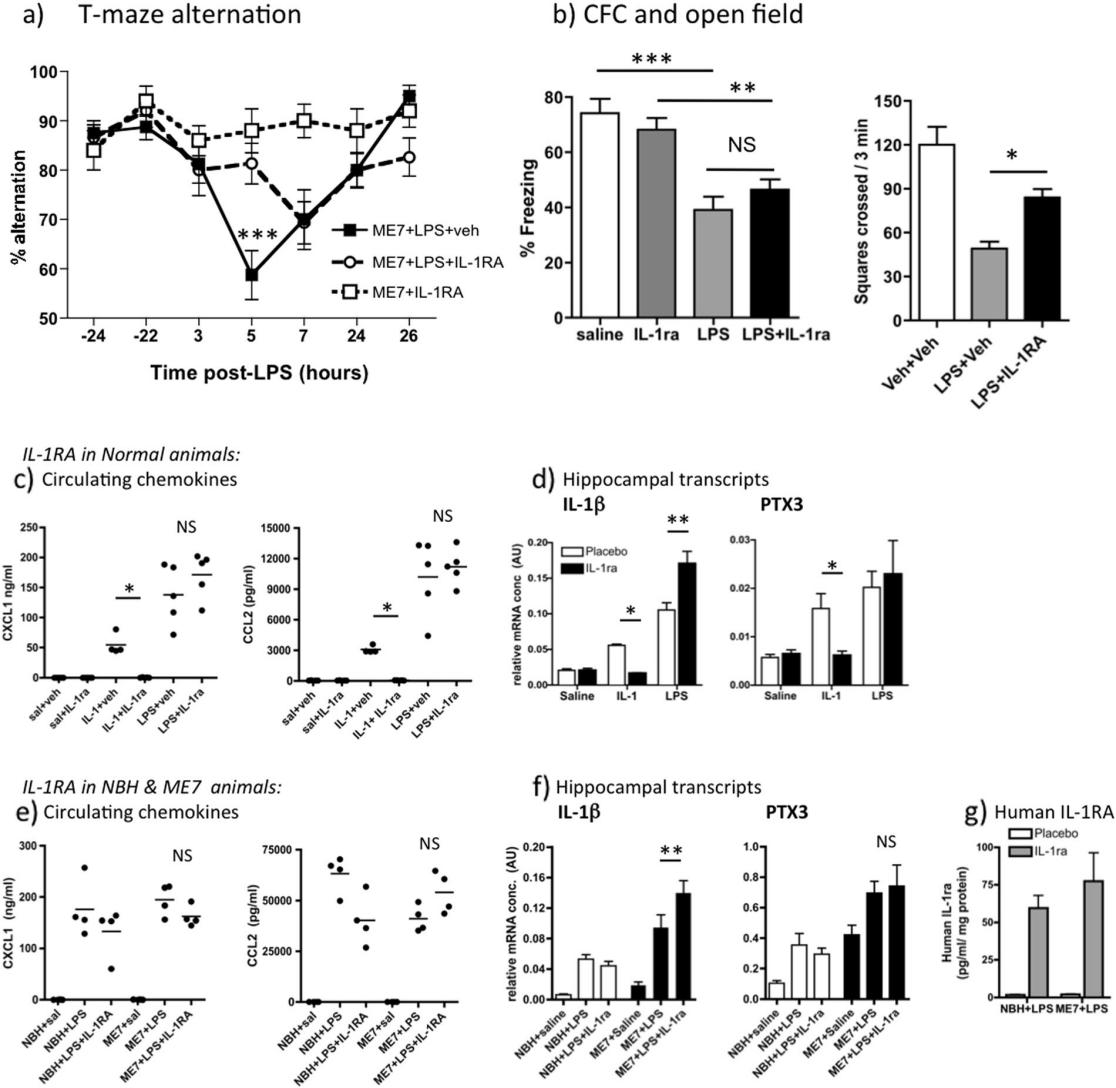
Dissociable effects of systemic IL-1 receptor antagonist (IL-1RA) on T-maze alternation and contextual fear conditioning. **a** Working memory performance of ME7 animals, 16 weeks post inoculation, challenged with LPS (100 μg/kg i.p.) in the presence or absence of IL-1RA (10 mg/kg i.p. immediately following LPS). These data arise from two independent experiments, totalling *n* = 16, except ME7 + IL-1RA controls (*n* = 10). Significant Bonferroni post hoc (*p* < 0.01) after significant two-way ANOVA denoted by ***. **b** Performance of normal mice in CFC (time spent freezing 48 h following 0.4 mA foot shock) and in open field activity following systemic challenge with saline or LPS (100 µg/kg i.p.), in the presence or absence of IL-1RA (10 mg/kg i.p.). All groups were *n* = 11 except WT + saline (*n* = 7) for CFC experiment and *n* = 5–7 for open field. Data are expressed as mean ± SEM and were analysed by two-way ANOVA. Significant differences, assessed by Bonferroni post hoc after significant ANOVA are denoted by **p < 0.01 and ***p < 0.001. **c** Effect of i.p. recombinant IL-1RA on plasma chemokine (CXCL1, CCL2) induction 2 h post administration of IL-1β (15 μg/kg, i.p.) or LPS (100 μg/kg, i.p.), analysed by ELISA. Bonferroni post hoc tests (after significant ANOVA) are annotated by *p < 0.05. **d** Expression of pro-inflammatory genes IL-1β and PTX3, examined in the hippocampus after the same IL-1β or LPS challenges ± IL-1RA. Significant differences by Bonferroni post hoc test are denoted by *p < 0.05 and **p < 0.01; *n* = 5 per group (except IL-1β + veh *n* = 4). **e** Effect of i.p. recombinant IL-1RA on CXCL1 and CCL2 induction 3 h post administration of LPS (100 μg/kg, i.p.) in NBH and ME7 animals (*n* = 4). Using IL-1RA and disease as between-subjects factors there was no main effect of treatment (*F*_1,12_ = 3.32, *p* = 0.1592) or no interactions (*F*_1,12_ = 0.06, *p* = 0.6651) for CXCL1 and no effect of treatment (*F*_1,12_ = 1.08, *p* = 0.3199) but a significant interaction between disease and IL-1RA for CCL2 (*F*_1,12_ = 13.39, *p* = 0.0033). **f** Expression of IL-1β and PTX3 in the hippocampus 3 h after LPS challenges ± IL-1RA (*n* = 4 for all groups except NBH + LPS, NBH + LPS + IL-1RA: *n* = 6). **g** Human IL-1RA was measured by human IL-1RA ELISA in hippocampal tissue of NBH and ME7 animals 3 h after treatment with LPS ± IL-1RA (*n* = 4 in all groups). All data are presented as mean ± SEM

Since acute cognitive impairments following systemic LPS were immediately preceded by robust circulating IL-1β, we hypothesised that systemic IL-1β contributed to these deficits and we therefore assessed whether peripheral treatment with the receptor antagonist IL-1RA would protect against the T-maze and CFC deficits described in Fig. 1. IL-1RA at 10 mg/kg (i.p.) significantly protected against LPS-induced T-maze deficits in ME7 animals in a time-dependent manner (Fig. 2a). There was a significant effect of treatment (*F*_2,234_ = 4.78, *p* = 0.139) and an interaction of treatment and time (*F*_12,234_ = 3.67, *p* < 0.0001) and Bonferroni post hoc comparison showed a highly significant difference between ME7 + LPS + IL-1RA and ME7 + LPS + veh at 5 h (*p* < 0.001). However, both LPS-treated groups were equally impaired at 7 h, so protection is robust at 5 h, when LPS-induced deficits peak, but does not last.

Conversely, systemic IL-1RA had no protective effect against the LPS-induced CFC deficit despite mitigating the effects of LPS on open field activity (Fig. 2b). LPS robustly induced decreased freezing in C57BL6 mice (main effect of LPS by two-way ANOVA, *F*_3,36_ = 38.68, *p* < 0.0001). Freezing, 48 h after co-treatment with LPS + IL-1RA (10 mg/kg i.p.), was not significantly different from that induced by LPS (Fig. 2a, no effect of drug: *F* = 0.08). Therefore peripheral IL-1RA is protective against LPS-induced working memory deficits and against suppression of open field activity but not against LPS-induced impairment of consolidation of contextual memory. Although these LPS-induced deficits become manifest across different timescales (i.e. 3–7 h for T-maze and at 48 h for CFC), LPS and IL-1β impair consolidation of contextual memory in the first hours after exposure to shock and context pairing [33, 36]. Therefore both T-maze and CFC tasks illustrate inflammation-induced disruption of cognitive processes in the hours directly after LPS administration, so it was necessary to apply IL-1RA during this period.

We considered it essential to confirm that i.p. IL-1RA (10 mg/kg) actually blocked IL-1β action so we assessed its ability to block well-recognised indices of systemic and central (hippocampal) inflammatory effects of LPS (100 μg/kg) and IL-1β (15 μg/kg). A range of pro-inflammatory mediators are induced across the timecourse 1–8 h post LPS or IL-1β [28, 37] and here we chose 2 h to capture the peak of most of these. Plasma was prepared 2 h post treatment with recombinant IL-1RA (10 mg/kg, i.p.) and simultaneous injection of IL-1β, LPS or saline. Both LPS and IL-1β increased plasma CXCL1 and CCL2. IL-1RA had no effect on LPS-induced circulating chemokine but completely blocked IL-1-induced chemokine (*p* < 0.05, Bonferroni post hoc after significant one way ANOVA, Fig. 2c). Hippocampal expression of pro-inflammatory genes IL-1β and PTX3 was also increased by both systemic LPS and IL-1β treatments (Fig. 2d). Once again IL-1-induced increases in both IL-1β and PTX3 were inhibited by systemic IL-1RA (*p* < 0.05), but this treatment failed to block, and in fact even enhanced, LPS-induced hippocampal increases in IL-1β and PTX3 (Fig. 2d). These data indicate that circulating LPS still alters plasma and brain inflammatory profiles despite successful inhibition of systemic IL-1β action: LPS signals to the brain despite absence of systemic IL-1β activity.

We also assessed whether effects of IL-1RA were different in ME7 by examining expression of these same indices of LPS and IL-1 actions in NBH and ME7 animals 3 h after treatment with LPS ± IL-1RA. As before, LPS robustly increased the circulating levels chemokines CXCL1 and CCL2 but IL-1RA had limited impact on these levels, with huge circulating concentrations remaining regardless of treatment (Fig. 2e). Two-way ANOVA analysis followed by Bonferroni post hoc analysis showed that IL-1RA did not reduce circulating chemokines in ME7 animals: There were no significant main effects or interactions for CXCL1, and while there was an interaction between disease and treatment for CCL2 (*F*_1,12_ = 13.39, *p* = 0.003), Bonferroni post hoc analysis showed that CCL2 was not different between ME7 + LPS and ME7 + LPS + IL-1RA.

Hippocampal expression of pro-inflammatory genes IL-1β and PTX3 was also examined in ME7 and NBH and disease-associated increases were apparent for both transcripts (as previously reported in ref. [38]): main effect of disease (*F*_1,16_ ≥ 22.85, *p* ≤ 0.0002). LPS produced significant increases in both transcripts and IL-1RA did not decrease these, and indeed IL-1β mRNA was significantly increased in ME7 + LPS animals when treated with IL-1RA (Bonferroni post hoc, *p* < 0.01). Therefore, systemic IL-1RA did not reduce hippocampal IL-1β mRNA. Collectively these data demonstrate that even though IL-1RA blocks circulating IL-1β actions, LPS still induces brain inflammatory activation, including IL-1β.

Finally we used anti-human IL-1RA ELISA on these in vivo tissues to assess whether peripherally applied human IL-1RA reaches the hippocampus, since it could be hypothesised that IL-1RA could show protective effects because of compromised blood brain barrier (BBB) allowing access of IL-1RA selectively to the ME7 hippocampus. Low levels of human IL-1RA were detectable in the hippocampus of all IL-1RA-injected animals but concentrations were equivalent in NBH and ME7 animals (Fig. 2g). These levels (60–70 pg/ml/mg protein) are almost 7500-fold lower than plasma levels (481,511 ± 95,460 pg/ml). These data indicate that (i) a tiny fraction of IL-1RA penetrates the brain parenchyma and (ii) that this is not significantly higher in neurodegeneratively diseased (ME7) animals. Moreover, although IL-1RA is known to have a short half-life [22, 39], the levels measured in the blood at 3 h post treatment show that IL-1RA remained approximately 2000-fold higher than circulating IL-1β at what is a key stage in inducing working memory dysfunction and contextual memory consolidation.

### IL-1β, TNF-α and redundancy in inflammation-induced cognitive dysfunction

**Fig. 3:**
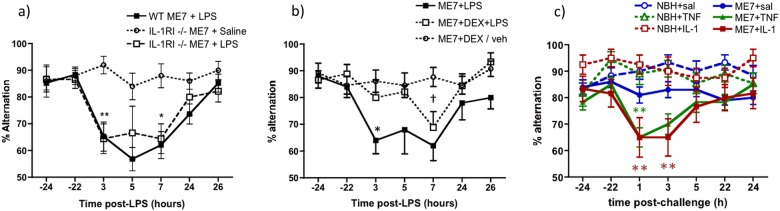
LPS-induced working memory deficits in ME7 animals are intact in IL-1R1^−/−^ mice but are blocked by inhibition of systemic cytokine synthesis and replicated by administration of IL-1β or TNF-α. **a** T-maze alternation, of WT and IL-1RI^−/−^ animals, 16 weeks post inoculation with ME7, in the presence or absence of systemic LPS challenge (100 μg/kg i.p.) was assessed for 10 trials 24 h before acute challenge, 15 trials 3–9 h post challenge and 10 trials 24 h after the challenge. WT ME7 + LPS and IL-1R1^−/−^ ME7 + LPS were not different at any time. Significant Bonferroni post hoc differences between IL-1R1^−/−^ ME7 + LPS and IL-1R1^−/−^ ME7 + saline are denoted by *p < 0.05 and **p < 0.01 (*n* = 10 except WT ME7 + LPS *n* = 19). **b** Working memory performance after systemic LPS (100 μg/kg i.p.) was assessed in the presence or absence of dexamethasone-21-phosphate (2 mg/kg i.p.). Significant post hoc differences between ME7 + LPS + veh and ME7 + LPS + DEX (by Bonferroni post hoc comparisons after a significant ANOVA) are denoted by *p < 0.01 and between ME7 + LPS + DEX and ME7 + DEX/veh by ^†^*p* < 0.01. IL-1R1^−/−^ ME7 + LPS *n* = 10, IL-1R1^−/−^ ME7 + Dex + LPS *n* = 9 and IL-1R1^−/−^ ME7 + Dex/vehicle *n* = 13. Data combined from two independent experiments. **c** Systemic administration of IL-1β (15 µg/kg) or TNF-α (50 µg/kg) induced acute working memory deficits in T-maze alternation. All animals were assessed for 10 trials 24 h before acute challenge, 15 trials 1–7 h post challenge and 10 trials 24 h after the challenge. Group sizes were as follows: NBH + saline (*n* = 16), NBH + TNF (*n* = 11), NBH + IL-1β (*n* = 14), ME7 + saline (*n* = 20), ME7 + TNF (*n* = 12) and ME7 + IL-1β (*n* = 12). Each cytokine was tested in two independent experiments. Data pertaining to TNF-α originally published in Hennessy et al. [44]. Significant main effects and interactions are described in the main text. Significant differences by Bonferroni post hoc analysis between ME7 + IL-1β and NBH + IL-1β (red) and between ME7 + TNF-α and NBH + TNF-α (green) are denoted by **p < 0.01. All data are presented as mean ± SEM

To further understand the contribution of IL-1 signalling to the acute working memory deficits induced by LPS in ME7 mice, we inoculated wild-type and IL-1R1^−/−^ mice with ME7 and, at 16 weeks into disease progression, challenged these mice with LPS (100 μg/kg i.p.) or saline (Fig. 3a). LPS treatment induced working memory impairments in IL-1R1^−/−^ ME7 animals (main effect of treatment: *F*_12,245_ = 16.61, *p* < 0.0001) but the deficits were not significantly different to those in wild-type ME7 + LPS animals at any time point (All Bonferroni post hoc tests *p* > 0.05, Fig. 3a). Thus, IL-1RI is not indispensable for LPS-induced acute working memory deficits in ME7-inoculated animals. While this may appear contradictory, IL-1RI^−/−^ mice also showed the normal appearance of LPS-induced sickness and weight loss (equivalent at 24 h post LPS in WT and IL-1R1^−/−^ mice: 3.22 g vs 2.96 g) and this is consistent with previous data showing that IL-1R1^−/−^ mice display the full spectrum of innate immune responses to LPS (http://jaxmice.jax.org/strain/003245.html; ref. [40]). To confirm this we assessed cFOS responses in known IL-1-responsive brain regions after systemic LPS (100 µg/kg i.p.). These data are shown in supplementary table 1. Despite this, systemic IL-1RA was still effective in reducing LPS-induced sickness behaviour (Fig. 2b). Collectively these data support the concept of significant redundancy of specific cytokines in coordination of inflammatory responses to LPS. Therefore, while IL-1 is indisputably important in inducing sickness behaviour responses (ref. [41] and Fig. 2b), and here systemic IL-1RA is protective against LPS-induced cognitive deficits, other cytokine systems can mediate roles of IL-1 when animals have developed in the absence of IL-1RI^−/−^ signalling.

To accommodate this redundancy in pro-inflammatory cytokine functions, we used dexamethasone-21-phosphate (DEX; 2 mg/kg) to block multiple LPS-induced pro-inflammatory cytokines and to assess its impact on LPS-induced T-maze impairments in IL-1R1^−/−^ ME7 mice (Fig. 3b). This dose was sufficient to block systemic secretion of IL-1β, TNF-α and IL-6 (Fig. S2 and ref. [29]). DEX alone had no significant impact on T-maze alternation. LPS induced working memory impairments in ME7 animals and this was significantly reduced in animals pre-treated with DEX (Fig. 3b). Repeated measures two-way ANOVA showed a main effect of treatment (*F*_2,30_ = 8.7, *p* = 0.001) and a treatment × time interaction (*F*_12,180_ = 2.28, *p* < 0.01). ME7 + LPS was significantly different to both DEX groups at 3 h (*p* < 0.01; Bonferroni), although ME7 + LPS and ME7 + LPS + DEX were no longer significantly different at 7 h. Thus DEX protects against LPS-induced deficits but some cognitive impairment eventually occurs despite DEX inhibition of systemic cytokine synthesis.

These data suggested that systemic cytokines contribute to LPS-induced working memory impairments. Therefore, we interrogated the roles of specific systemic cytokines. We treated animals with IL-1β (15 µg/kg i.p.) and assessed for working memory deficits. Moreover, it has been demonstrated that in IL-1RI^−/−^ mice, TNF-α compensates for the lack of IL-1 signalling and mediates sickness behavioural and weight loss responses to LPS [42]. Therefore, in separate experiments, the impact of systemically administered TNF-α on T-maze performance was assessed in NBH and ME7 animals. Impairments were not produced by either cytokine in NBH animals but both IL-1β and TNF-α challenges rapidly induced acute working memory deficits in ME7 animals that had largely resolved by the 5–7 h timepoint (Fig. 3c). The IL-1β experiment showed an interaction of disease and treatment (*F*_1,53_ = 5.42, *p* = 0.0236) and planned Bonferroni pairwise comparisons showed significant differences between ME7 + saline and ME7 + IL-1β at 1 and 3 h and between ME7 + saline and ME7 + TNF-α at 1 h (*p* < 0.05). As previously described [43] ME7 + TNF-α animals were significantly different from both NBH + TNF-α and from ME7 + saline at 1 h after a significant interaction of disease, treatment and time by three-way ANOVA (*F*_6,328_ = 3.09, *p* < 0.01). Thus, systemic IL-1β and TNF-α cause acute and transient working memory deficits in animals with prior neurodegenerative pathology but have no effect on working memory in normal animals. These deficits are somewhat milder than those induced by LPS and recovery is apparent by 5 h.

### Neuronal sensitivity to IL-1β

Since equivalent systemic IL-1 levels produce differential cognitive outcomes in ME7 and NBH animals, we predicted that neurons of the degenerating brain might also be more sensitive to equivalent concentrations of IL-1. To address this hypothesis we applied IL-1β directly to ex vivo hippocampal slices and performed whole-cell patch clamp recordings from CA1 pyramidal cells from NBH and ME7 animals at 18–19 weeks post inoculation.

CA1 pyramidal neurons from ME7 animals had a significantly depolarised resting membrane potential (*V*_m_) compared to those from NBH animals (−61 ± 2 mV in NBH and −51 ± mV in ME7, *n* = 13 cells from five NBH animals and 24 cells from eight ME7 animals, unpaired *t*-test, *p < 0.0001, Fig. S3).

**Fig. 4:**
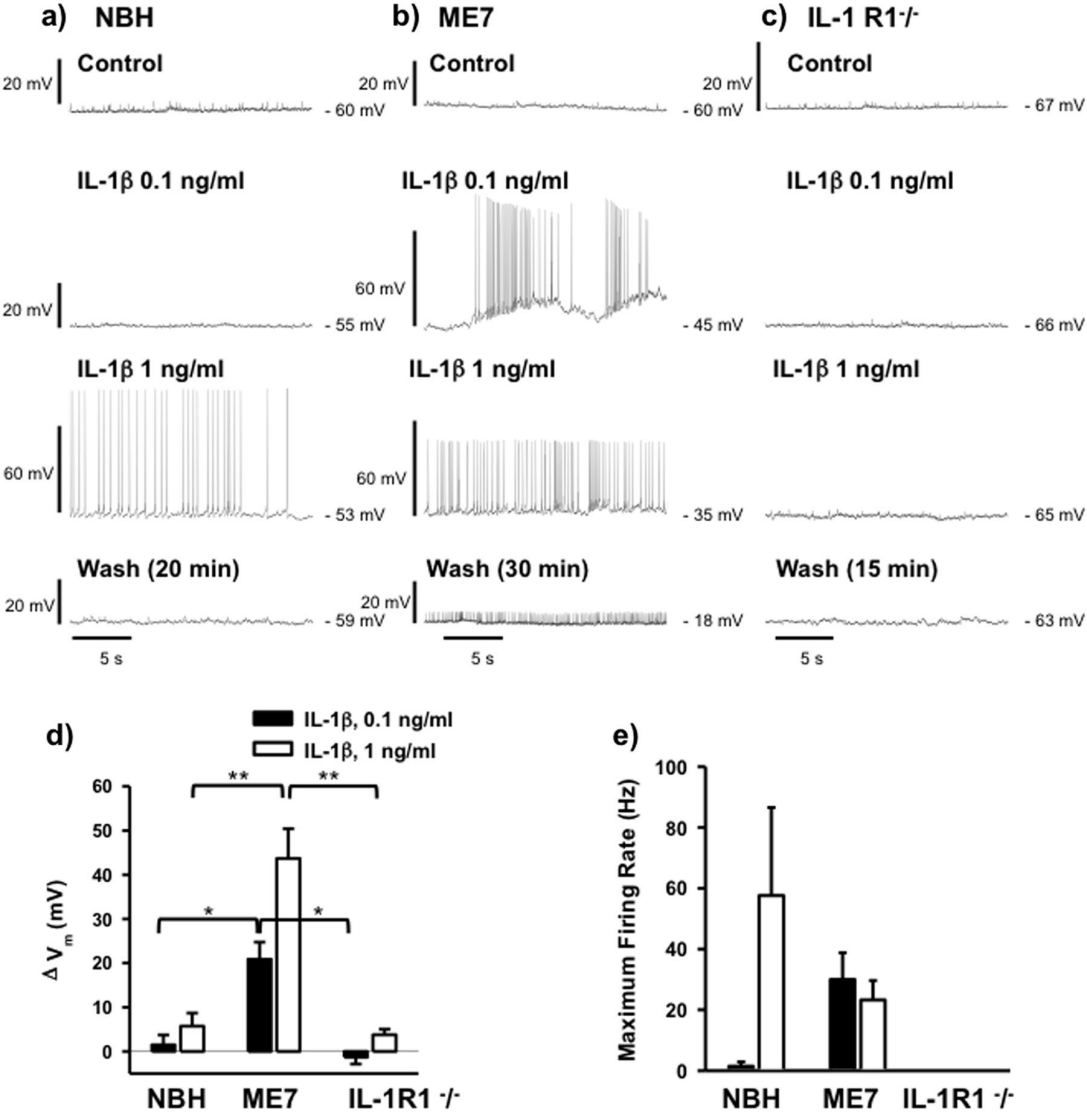
Differential sensitivity of CA1 pyramidal cells to IL-1β in hippocampal slices from NBH and ME7 animals. Traces illustrating current clamp recordings from CA1 pyramidal cells from NBH (**a**), ME7 (b) and IL-1RI^−/−^ (**c**) animals during control, baseline recordings and in the presence of IL-1β at concentrations of 0.1 and 1 ng/ml and following washout. The washout period was prolonged in ME7 animals (30 min) in order to maximise the chance of observing a return to resting membrane potential, but this depolarisation appeared irreversible. The effects of IL-1β were quantified by measuring the change in baseline membrane potential (Δ*V*_m_), averaged over 10 s, (**d**) and changes in the maximum action potential firing rate (**e**). Neurons from ME7 animals were more sensitive to IL-1β, exhibiting greater depolarisation of *V*_m_ in response to IL-1β at either concentration and a much higher firing rate at the lower concentration of 0.1 ng/ml (*n* = 5 cells from three NBH animals, *n* = 13 cells from five ME7 animals for 0.1 ng/ml and 5 cells from three ME7 animals for 1 ng/ml). No action potential firing was induced in cells from IL-1 R1^−/−^ animals at either concentration of IL-1β tested (*n* = 4 cells from two animals). Statistically significant differences between different animal groups treated with 0.1 ng/ml and different animal groups treated with 1 ng/ml are assessed are denoted by *p < 0.05 or **p < 0.01 (Bonferroni post hoc tests after significant one way ANOVAs). All data are mean ± SEM

We then tested the effects of bath application of IL-1β while recording from cells in current clamp mode, using current injection adjusted to maintain membrane potential at −60 mV during baseline recordings (Fig. 4a–c). Neurons from ME7 animals were significantly more sensitive to IL-1β, which depolarised resting membrane potential by 21 ± 4 mV at 0.1 ng/ml in ME7 animals (*n* = 13 cells from five animals) but had little effect in NBH animals (1.5 ± 2 mV; *n* = 5 cells from three animals; *p* = 0.013, Bonferroni post hoc, Fig. 4d). At 1 ng/ml, IL-1β depolarised ME7 animals’ resting membrane potential by 44 ± 7 mV (*n* = 5 cells from three animals) but also had milder effects in NBH animals (6 ± 3 mV; *n* = 5 from three animals; *p* < 0.001, Bonferroni post hoc, Fig. 4d). ME7 were thus, significantly more sensitive to IL-1β-induced depolarisation at both IL-1β concentrations tested.

As a consequence, IL-1β induced action potential firing in ME7 CA1 neurons at 0.1 ng/ml, with a maximum firing rate of 30 ± 9 Hz (0.1 ng/ml, *n* = 13 cells) but did not do so in NBH animals (Fig. 4e). At 1 ng/ml, IL-1β induced spiking at maximum rates of 23 ± 6 Hz (1 ng/ml, *n* = 5 cells, Fig. 4e) in ME7 animals and at a highly variable maximum rate of 58 ± 29 Hz in NBH neurons (*n* = 5 cells, Fig. 4e). The lower firing rates in ME7 cells can be attributed to the greater depolarisation induced by IL-1β, acting to increase sodium channel inactivation and therefore limiting spike firing frequency. Depolarisation of ME7 neurons persisted even after a more prolonged washout of IL-1β, with cells becoming sufficiently depolarised to inhibit further action potential firing, suggesting that exposure to IL-1β had an irreversible, detrimental effect on these cells.

The effect of IL-1β on ME7 neurons does not appear to be mediated by modulation of excitatory synaptic transmission since 1 ng/ml IL-1β had no effect on evoked excitatory postsynaptic currents (EPSC amplitude 105 ± 32% of control amplitude, *n* = 4 cells from two ME7 animals, Supplementary Fig. S4). The ability of IL-1β to induce spiking activity in CA1 neurons was dependent on IL-1R1 expression since IL-1β had little effect on resting membrane potential and did not induce action potential firing in slices from IL-1R1^−/−^ animals (*n* = 4 cells from two animals, Fig. 4c–e). Moreover, TNF-α (20 ng/ml) had no effect on membrane potential in slices from IL-1R1^−/−^ animals (−64 ± 2 mV in control and −64 ± 2 mV in TNFα, *n* = 5 cells from two animals, Supplementary data, S5).

### Consequences of exaggerated IL-1 responsiveness: apoptosis

**Fig. 5:**
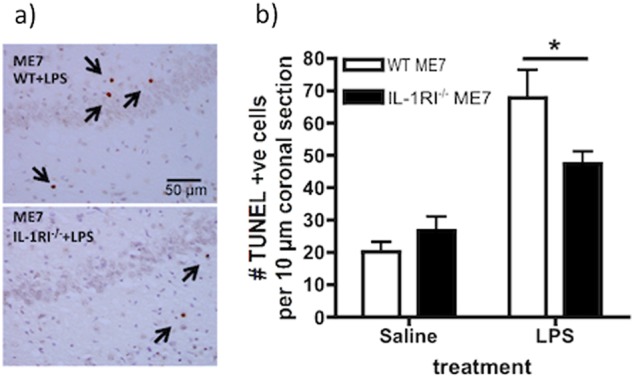
IL-1RI-dependence of LPS-induced apoptosis. ME7 animals, on a wild-type or IL-1R1^−/−^ background, were challenged i.p. with LPS (750 µg/kg) or saline. **a** Representative hippocampal fields (strata radiatum, pyramidal and oriens at the CA1/subiculum border) after TUNEL immunohistochemistry for apoptotic cells at 18 h post LPS or sterile saline, with condensed TUNEL-positive cells labelled with arrows. Scale bar = 50 µm. **b** Apoptotic cells were counted in 10 µm coronal sections. Data are shown as mean ± SEM with *n* = 5 in all groups. * denotes ME7 + LPS significantly different to ME7 IL-1R1^−/−^ + LPS (*p* < 0.005) by Bonferroni post hoc after significant interaction between strain and treatment by two-way ANOVA (*F* = 6.05, df 1,16; *p* = 0.0257)

Since neurons in the diseased brain were more sensitive to the effects of IL-1 and failed to recover their resting membrane potential, we hypothesised that the previously reported neuronal apoptosis induced by systemic LPS [25] would be mediated by IL-1RI. ME7 animals were challenged with LPS (750 µg/kg i.p.) and euthanised 18 h later, the time at which LPS-induced neuronal apoptosis has previously been demonstrated [25]. TUNEL labelling was performed to assess for apoptotic cell death in 10 µm coronal sections. LPS induced robust apoptosis in wild-type ME7 animals, with respect to ME7 + saline animals and the number of apoptotic cells was significantly reduced in IL-1R1^−/−^ ME7 + LPS animals (Fig. 5a, b). Two-way ANOVA showed a main effect of treatment (*F* = 38.59, df 1,16 *p* < 0.0001) and an interaction of treatment and genotype (*F*_1,16_ = 6.05, *p* = 0.026) and Bonferroni post hoc comparison showed that ME7 + LPS was significantly more affected than ME7 IL-1R1^−/−^ + LPS (*p* < 0.05) and ME7 + saline (*p* < 0.001), although LPS still increased apoptosis somewhat in IL-1RI^−/−^ (*p* < 0.05). Therefore, LPS-induced apoptosis in ME7 animals is partially mediated by IL-1RI. Our prior studies had established that NBH animals were not similarly susceptible to LPS-induced apoptosis [25]. However, we have also verified that LPS did not produce acute apoptosis in NBH animals under the current conditions (Supplementary Figure S6). Similarly, the exaggerated hypothermic response of ME7 animals to systemic LPS was also mediated by IL-1RI (Fig. S7).

## Discussion

We have demonstrated that systemic inflammation impairs cognitive and neuronal function in multiple ways, by dissociable IL-1-dependent mechanisms. While LPS had no impact on working memory in normal animals it robustly impaired working memory in animals with existing neurodegenerative pathology. Systemic LPS-induced working memory deficits were mediated by circulating IL-1β. Systemic LPS also induced IL-1R1-dependent apoptotic cell death in the neurodegenerating brain and CA1 neurons in hippocampal slices showed heightened vulnerability to IL-1β-induced, non-reversible, loss of membrane potential. The data suggest that systemic inflammation induces both transient cognitive dysfunction and lasting brain injury and that IL-1 contributes to both processes, but by dissociable mechanisms. Since systemic inflammation can trigger episodes of delirium in susceptible individuals [9] and episodes of delirium interact synergistically with existing cognitive impairment to accelerate dementia progression [44], understanding the mechanistic basis of both the acute deficits and the lasting injury is of crucial importance. The current data adds to our knowledge on transient and lasting effects of systemic inflammation on the already degenerating brain.

### Dissociations in inflammation-induced, hippocampal-dependent cognitive impairment

Although impairment of memory consolidation in the CFC paradigm is the best characterised effect of systemic inflammation on cognitive function in young healthy rodents (whether induced by LPS, infection or surgical trauma) [17, 20, 21], we have argued that this consolidation of long-term memory may not be optimal for studying the dynamic and fluctuating attentional and working memory deficits observed during delirium [45]. Hippocampal IL-1β clearly impairs late-phase LTP [46] and impairs consolidation of contextual memory both in young and aged LPS/*E. coli*-treated rats [47] and in post-operative mice [20]. However, the consolidation of memory that is central to CFC is associated with impairment in late-phase LTP [48] and deficits in this relatively slow, protein synthesis-dependent, process cannot explain the disruption in dynamic short-term memory and/or attentional processes described here. There is no intuitive role for memory consolidation in the current T-maze task and the neurological substrates required for CFC and T-maze alternation are manifestly different [49]. Here we show that working memory and contextual memory consolidation are differently affected by systemic inflammation: despite the CFC impairment observed with 100 µg/kg LPS in the current study, neither this nor 200 µg/kg LPS was sufficient to impair working memory in the T-maze in normal animals. Therefore systemic inflammation differentially affects different hippocampal-dependent tasks, as has previously been suggested from context discrimination tasks [32, 50]. This dissociation demonstrates that one cannot assume that all IL-1 β effects on cognition are mediated by the same mechanism and research into IL-1β effects on cognitive function must interrogate multiple mechanisms to explain deficits in different cognitive domains.

The same LPS dose that failed to impair working memory in normal animals did induce robust working memory deficits in animals with prior hippocampal synaptic loss. These deficits were ameliorated by peripheral treatment with IL-1RA, but this treatment had no beneficial effect with respect to the CFC deficits already described. Although the LPS-induced deficits become manifest across different timescales (i.e. 3–7 h for T-maze and at 48 h for CFC), it has been clearly demonstrated that LPS and IL-1β impair consolidation of contextual memory and that this occurs in the hours after exposure to shock and context pairing [33]. Treatment after consolidation has been completed, but directly before testing of retention, does not impair memory [32]. Therefore the cognitive process that LPS is impairing in the CFC task occurs in the hours immediately after exposure to the context. Therefore both T-maze and CFC tasks experience inflammation-induced disruption of cognitive processes in the hours directly after LPS administration and it is this rather than the time at which they are tested that is relevant to determine the appropriate time of application of treatments in order to intervene in these processes. These data illustrate that systemic inflammation produces impairments in multiple cognitive domains and that, at least with respect to these two hippocampal-dependent tasks, there are dissociable IL-1-dependent mechanisms. IL-1β is described to act centrally to impair consolidation in LPS-induced CFC deficits [19–23], but appears to act systemically here to impair working memory, as we discuss below.

### Systemic versus central effects

That the working memory impairment induced by LPS is mediated by circulating rather than centrally produced IL-1β is supported by multiple strands of evidence. (1) Peripherally applied IL-1RA was protective against the working memory deficits and CNS concentrations were 1/7500 of levels in the plasma at the time of protection against the deficits, consistent with prior data that peripherally administered IL-1RA (17 kDa) shows limited BBB penetration [51]. Moreover, although IL-1RA entry to the brain is thought to occur primarily when and where the BBB has been breached [39] we observed similar levels in NBH and ME7 animals arguing against the idea that existing neurodegenerative disease made the brain more permeable to IL-1RA (2) Cytokine and chemokine analysis shows that IL-1RA blocked IL-1 action in the blood (Fig. 2c) but did not prevent LPS-induced changes in hippocampal inflammatory transcripts, including de novo IL-1 in the brain. This is consistent with data showing direct activation of the brain vasculature by systemic LPS [52] and CNS inflammatory mediator production occurring despite abrogation of systemic cytokines via dexamethasone-21-phosphate inhibition [30] or by depletion of peripheral TLR4-positive macrophages [6, 53–55]. (3) DEX was 90% effective in reducing systemic IL-1β synthesis but not at all effective at blocking CNS transcription and microglial synthesis of IL-1β (Fig. S2 and ref. [30]). (4) Systemic administration of IL-1β is sufficient to induce the same deficits as LPS. Thus IL-1RA appears to act peripherally rather than centrally to block IL-1 action with respect to working memory.

### Time-dependent protection and redundancy in inflammation-induced impairments

The protection afforded by IL-1RA is temporary (Fig. 2a). IL-1RA remained at 481.5 ng/ml at 3 h post injection in the current study, compared to 125 pg/ml plasma IL-1β 2 h post LPS at 100 µg/kg [37]. Therefore, with a stoichiometric excess of greater than 2000-fold over IL-1β, we are confident that the known short half-life of IL-1RA was not sufficient to prevent biologically relevant levels to block effects of circulating IL-1 at a key time in memory disruption. However, it is possible that the rapid renal metabolism of IL-1RA [56] could have caused levels to fall below therapeutic efficacy [22] during the later trials (7–9 h post LPS). The protection offered by dexamethasone administration in IL-1R1^−/−^ animals also waned at 7 h post challenge. Given that CNS inflammatory mediator production persisted even in the presence of systemic IL-1RA or dexamethasone (Fig. S2), it is plausible that propagation of central mediators such as IL-1β may have additional effects, or expression of additional inflammatory mediators sufficient to disrupt cognition, may also have occurred independent of systemic IL-1β. One such candidate, which may contribute to systemic IL-1-independent effects, is TNF-α. We show here that systemically administered TNF-α was sufficient, alone, to produce acute impairments and is robustly expressed after systemic challenge with LPS [28] and after inflammatory trauma such as the tibial fracture used in POCD models [20, 23]. The ability of TNF-α to mimic the effects of IL-1 is important in the light of LPS’ propensity to produce equivalent T-maze impairments in wild-type and in IL-1R1^−/−^. IL-1RI^−/−^ mice develop in the absence of IL-1 signalling and, despite the acknowledged importance of IL-1 in innate immune and sickness behaviour responses to LPS (ref. [41] and Fig. 2), these mice are known to show normal responses to systemic LPS (http://jaxmice.jax.org/strain/003245.html) [40]. Other cytokines, such as TNF-α, demonstrably compensate for the lack of IL-1 signalling to induce sickness behaviour responses in these mice [42]. Here, reducing systemic cytokines by >90% using DEX [29], produced robust, although temporally restricted, protection against LPS-induced working memory deficits and both TNF-α and IL-1 were sufficient to mimic LPS effects.

It remains unclear exactly how systemic IL-1 (or TNF-α) alters cognitive function in the current paradigm. Based on its rich expression of IL-1RI [57–59], the brain endothelium is an obvious first target and indeed we investigated and refuted the hypothesis that endothelial COX2 mediates this LPS-induced working memory deficit: we found that non-selective COX inhibitors protected against both LPS and IL-1-induced T-maze deficits, but only COX-1 inhibitors protected against LPS-induced T-maze deficits [60]. The rich expression of IL-1 receptors on hippocampal neurons [61] offers compelling support for the direct effect of locally produced IL-1 on memory function and LTP, but this is most relevant for the IL-1-dependence of CFC deficits shown by several previous authors [19, 20, 36]. The demonstration here that the CFC and T-maze tasks are dissociable on a number of levels emphasises that IL-1 exerts effects on different types of memory function through different mechanisms and here, the data support the idea of IL-1 acting first on targets outside the brain. IL-1 can also act directly on vagal afferents or on neurons proximal to the circumventricular organs, which lack a patent blood brain barrier. It is known that peripheral IL-1β can act directly on these neurons, which are not directly responsive to LPS [53], to induce expression of the immediate early gene cFOS here and in the amygdaloid complex [62, 63]. Similarly IL-1 has robust effects on peripheral energy metabolism [64] which may have multiple effects on brain function. Ultimately it will be important to dissect the precise role of IL-1 in this paradigm using cell-specific knockouts of IL-1RI and this is an important target for future experiments.

### Direct effects of IL-1β on hippocampal neurons

Despite equivalent systemic inflammation in NBH and ME7 animals, differential CNS outcomes were observed in ME7 animals and this may occur in several different ways. Firstly, the degenerating brain is ‘primed’ to show exaggerated CNS IL-1β responses to systemic LPS [25, 65–67]. This exaggerated CNS IL-1 has been assumed to be responsible for the exaggerated sickness behaviour responses to systemic LPS observed in aged and ME7 prion-diseased animals [65, 68] and here we demonstrated that exaggerated hypothermia induced by LPS in ME7 animals is very much mitigated in IL-1R1^−/−^ mice, confirming that IL-1 is a major driver of this heightened sickness response (Fig. S7).

Secondly, neurons in the diseased brain may be more susceptible to the effects of inflammatory mediators, and concentrations not deleterious to neuronal function in healthy individuals might disrupt function in diseased neurons. Here we ‘by-passed’ microglial priming by applying equal IL-1β concentrations directly to ex vivo hippocampal slices. IL-1β at 0.1 ng/ml (5.9 pM) had no effect on CA1 neurons from NBH animals but was sufficient to depolarise and induce maximal action potential firing in ME7 CA1 neurons. These diseased CA1 neurons were significantly more sensitive (low pM) than prior studies of IL-1-induced depolarisation (1 nM: [69, 70]) and the depolarisation observed appeared to be non-synaptic, in that IL-1 had no effect on evoked EPSCs or IPSCs (Fig. S4). These effects of IL-1β on CA1 neurons in ME7 animals likely disrupt the precise firing patterns of CA1 pyramidal cells that underlie the rate and temporal codes mediating hippocampal information processing and could contribute to acutely compromised function in multiple hippocampal-dependent tasks. The ex vivo CA1 spiking activity was dependent on IL-1RI^−/−^ and the observation that IL-1β had an irreversible, detrimental effect on CA1 cells from ME7 animals also suggests a role for hippocampal IL-1β in systemic inflammation-induced neuronal death. Increased neuronal death after systemic LPS in ME7 animals was independent of circulating IL-1β [30] and here we show that this LPS-induced apoptotic cell death was significantly reduced in IL-1R1^−/−^ mice. Although the specific target of LPS-induced IL-1 in producing cell death was not identified here, we propose that the microglial IL-1β shown here and previously, directly targets proximal neurons and contributes to loss of viability. Consistent with this, IL-1β applied directly to ME7 neurons, was sufficient to produce irreversible depolarisation and this effect was IL-1RI^−/−^ dependent. Thus we present evidence for mechanistic dissociation whereby systemic IL-1β has significant effects on working memory while central IL-1β has significant effects on neuronal integrity in the vulnerable brain.

The mechanisms by which IL-1 leads to non-reversible membrane depolarisation require further study. IL-1 is widely reported to have pro-convulsant activity and there are data supporting tyrosine kinase phosphorylation of NMDA receptor subunits [71, 72], contributing to IL-1-dependent neuronal death. Recently, altered IL-1R1 accessory protein (IL1RAcP) isoform expression in the aged brain was reported to underpin exaggerated effects of IL-1 in the hippocampus [73] it is important to investigate whether this mechanism might contribute to decreased viability of neurons in the degenerating brain upon acute elevations of IL-1.

It is a limitation of this study that higher doses of LPS were used to produce new apoptosis in the brain, than those used to produce working memory dysfunction. Our experience with this model over several studies has been that 100 µg/kg LPS is insufficient to produce measurable new pathology (no significant apoptosis or synaptic loss) or to alter the trajectory of disease (all animals return to baseline performance after acute deficits) [9, 74]. Conversely higher doses of LPS, which do alter pathology and change trajectory of disease [25, 30, 74], produce acute sickness that is ethically and scientifically incompatible with cognitive testing during the inflammatory episode.

## Conclusion

Systemic IL-1β drives acute working memory dysfunction but IL-1β also has robust direct effects on hippocampal neurons, leading to hyperexcitation and irreversible loss of membrane potential. This may provide a mechanistic explanation of LPS-induced IL-1RI-dependent cell death in the brain. The data support the idea that the acute and reversible cognitive deficits (including delirium) caused by systemic inflammation may operate via different mechanisms to the concurrent acute brain injury that presumably contributes to the negative long-term cognitive outcomes for patients after recovery from acute complications. Given that delirium contributes to progression of dementia, without necessarily exacerbating classical CERAD features like amyloid-β and Tau [11, 44] it is crucial to understand the extent to which delirium and acute brain injury occur by overlapping or dissociable mechanisms. The relevance of the current model for such investigations has been recently validated with respect to DSM-IV criteria and to alternative models [9, 75].

## Electronic supplementary material

Supplementary material
