# Correction: Acute transient cognitive dysfunction and acute brain injury induced by systemic inflammation occur by dissociable IL-1-dependent mechanisms

**DOI:** 10.1038/s41380-019-0403-7

**Published:** 2019-03-19

**Authors:** Donal T. Skelly, Éadaoin W. Griffin, Carol L. Murray, Sarah Harney, Conor O’Boyle, Edel Hennessy, Marc-Andre Dansereau, Arshed Nazmi, Lucas Tortorelli, J. Nicholas Rawlins, David M. Bannerman, Colm Cunningham

**Affiliations:** 1 School of Biochemistry and Immunology & Trinity College Institute of Neuroscience, Dublin 2, Ireland; 2 Department of Physiology, Trinity College Dublin, Dublin 2, Ireland; 3 Department of Experimental Psychology, University of Oxford, South Parks Road, Oxford, UK

**Keywords:** Neuroscience, Physiology, Psychology

**Correction to: Molecular Psychiatry**

10.1038/s41380-018-0075-8

published online 06 June 2018

Following publication of this article, the authors noticed an error in the abstract, where they incorrectly stated that: “Direct application of IL-1β to ex vivo hippocampal slices induced non-synaptic depolarisation and irreversible loss of membrane potential in CA1 neurons from diseased animals and systemic LPS increased apoptosis in the degenerating brain, in an IL-1RI−/−-dependent fashion”. This has now been corrected to: “Direct application of IL-1β to ex vivo hippocampal slices induced non-synaptic depolarisation and irreversible loss of membrane potential in CA1 neurons from diseased animals and systemic LPS increased apoptosis in the degenerating brain, in an IL-1RI-dependent fashion”. The authors would like to apologise for this error.

This has been corrected in both the PDF and HTML versions of the article.

