# Supplementary material for: Acute transient cognitive dysfunction and acute brain injury induced by systemic inflammation occur by dissociable IL-1-dependent mechanisms
Source: Mol Psychiatry. 2018 Jun 6;24(10):1533–48. doi: 10.1038/s41380-018-0075-8 (PMC6510649; doi:10.1038/s41380-018-0075-8)
Supplement: Supplementary file 1 — Supplementary material [file 41380_2018_75_MOESM1_ESM.docx]

**Supplementary material**

*Methods*

*Working memory: Food-rewarded and escape from water T-maze alternation tasks*

We assessed short term/working memory (3-9 hours post-LPS and 1-7 hours post-IL-1β) using alternation behaviour in both food-rewarded and escape-from-water-motivated T-maze tasks. We had specifically designed an ‘escape from shallow water’ T-maze task to allow assessment of working memory performance in animals experiencing sickness behaviour, as previously described ([Murray *et al.*, 2012](#_ENREF_48)). The T-maze was constructed of black perspex with the following dimensions (cm): long axis 67, short axis 38, depth 20 and arm width 7. There was a single 40 mm diameter hole at the end of each choice arm, 2 cm from the floor. Black exit tubes were inserted into these holes (these may also be blocked to prevent exit). A ‘guillotine’ door was inserted at the entrance to the choice arms to prevent access to one or other choice arm. This maze was filled with water at 20^o^C to a depth of 2 cm to motivate mice to leave the maze by “paddling” or walking “on tip-toe” to an exit tube.

Animals were taken with their cage mates to a holding cage. Each mouse was placed, individually, in the start arm of the maze with one arm blocked such that they were forced to make a left (or right) turn, predetermined by a pseudo-random sequence (equal numbers of left and right turns, no more than 2 consecutive runs to the same arm). On making this turn the mouse could escape from the water by entering the small tube, and then a transit tube, in which it was carried to another holding cage. The mouse was held here for 25 seconds (intra-trial interval) during which time the guillotine door was removed and the exit tube was switched to the alternate arm. The mouse was then replaced in the start arm and could choose either arm. The mouse must alternate (i.e. non-match to place) from its original exit arm to escape. On choosing correctly mice escape to the transit tube as before and are returned to their home cage. On choosing incorrectly the mice were allowed to self-correct to find the correct exit arm. Animals were trained for sessions of ten trials (inter-trial interval of 20 minutes) until reaching the criterion of performance of 70% or better (≥ 2 consecutive days), and not showing any evidence of a side preference (i.e. all errors occuring on the same side might indicate a preference for a particular body turn). This level of performance was maintained by running animals 3 times per week until reaching 15 to 16 weeks post-inoculation. At this time, those animals still at criterion (on average about 5% of animals fail to reach criterion) were challenged with LPS, IL-1β (15 µg/kg), TNF-α (50 µg/kg) or saline ± anti-inflammatory interventions.

Naive animals were also trained on an appetitively-motivated version of this task using an enclosed T-maze of the same dimensions. Mice were rewarded on sample and choice runs with 70µl of sweetened condensed milk contained in the lid of a 1.5 ml Eppendorf tube, placed at the end of the rewarded arm. Animals were food-deprived to approximately 90-95 % of free-feeding weight for this experiment. Animals choosing the correct arm were allowed to consume the entire reward while those choosing the incorrect arm were removed from the maze without reward and returned to the home cage. After successful baseline performance at above 80% alternation for more than 2 consecutive days, animals were treated with LPS (100 or 200 µg/kg) or with sterile saline and tested for 10 trials in a pseudorandomised sequence with equal numbers of left and right turns, as described for the water T-maze above.

*Immunohistochemistry*

Animals were transcardially perfused with heparinised saline followed by 10 % neutral buffered formalin (Sigma, Poole, UK) 12-15 minutes. Brains were removed and post-fixed for 3 days before embedding in paraffin wax. Coronal sections (10 μm) of brains were cut on a Leica RM2235 Rotary Microtome (Leica Microsystems, Wetzlar, Germany) at the various positions along the anterior-posterior axis from Bregma and floated onto electrostatically charged slides (Menzel-Glaser, Braunschweig, Germany) and dried at 37°C overnight. All sections were quenched for 20 minutes in 1% hydrogen peroxide in methanol, microwaved in citrate buffer (pH 6) for 2x5 minutes and blocked in 10% normal serum for 1h (and with 1% triton for 15 minutes for cFOS labeling). Primary antibodies were used as follows: mouse anti-cFOS (1/250, Santa Cruz), rabbit anti-IL-1β (1/50 in 20% normal goat serum, Peprotech). They were incubated overnight at 4^o^C. Sections were then incubated in appropriate biotinylated secondary antibody at 1/100 (Vector Labs) and developed using the ABC method with hydrogen peroxide and diaminobenzidine (DAB) as substrate.

*Electrophysiology*

For electrophysiological experiments using a Cs-based intracellular solution CsMeSO_4_ was substituted for KMeSO_4_ and CsCl for KCl in the patch pipette solution. Cells were voltage-clamped at -60 mV and input resistance and membrane capacitance were measured in response to a 10 mV depolarising voltage pulse.

**Results**

*Contextual fear conditioning in ME7 animals.*

The time spent freezing 48 hours post-conniditiong in the CFC paradigm was recorded for 5 minutes in NBH and ME7 animals (n=5) 16 weeks post-inoculation. There were no significant differences between NBH and ME7, although ME7 showed a modest, non-statistically significant effect (t-test: p=0.159). When LPS (100 µg/kg i.p) was administered to NBH and ME7 animals, it had equivalent effects on consolidation of contextual memory. Figure S1 demonstrates that all LPS animals showed a reduction in freezing compared to saline controls. Data were analysed by two-way ANOVA with treatment and disease as factors. There was a significant effect of LPS treatment (F_1,16_=34.75, p<0.0001) but no significant effect of disease (F_1,16_=1.94, p=0.1826) or interaction of these two factors (F_1,16_=0.82, p=0.3789). Therefore LPS impairs contextual memory equally in ME7 and NBH animals. This demonstrates another dissociation between CFC and working memory: despite the observation that working memory deficits are significantly worse in ME7+LPS compared to NBH+LPS, this is not the case for contextual memory consolidation.

**Supplementary Figure 1**


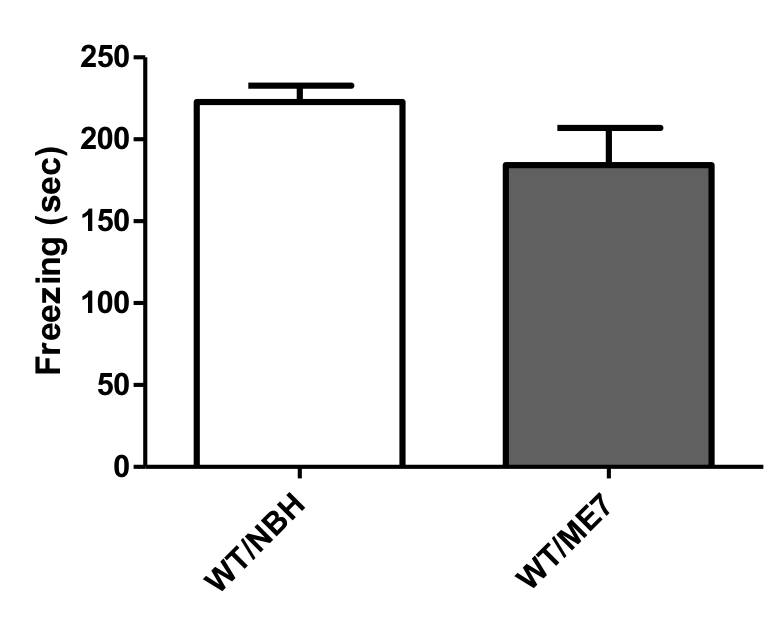

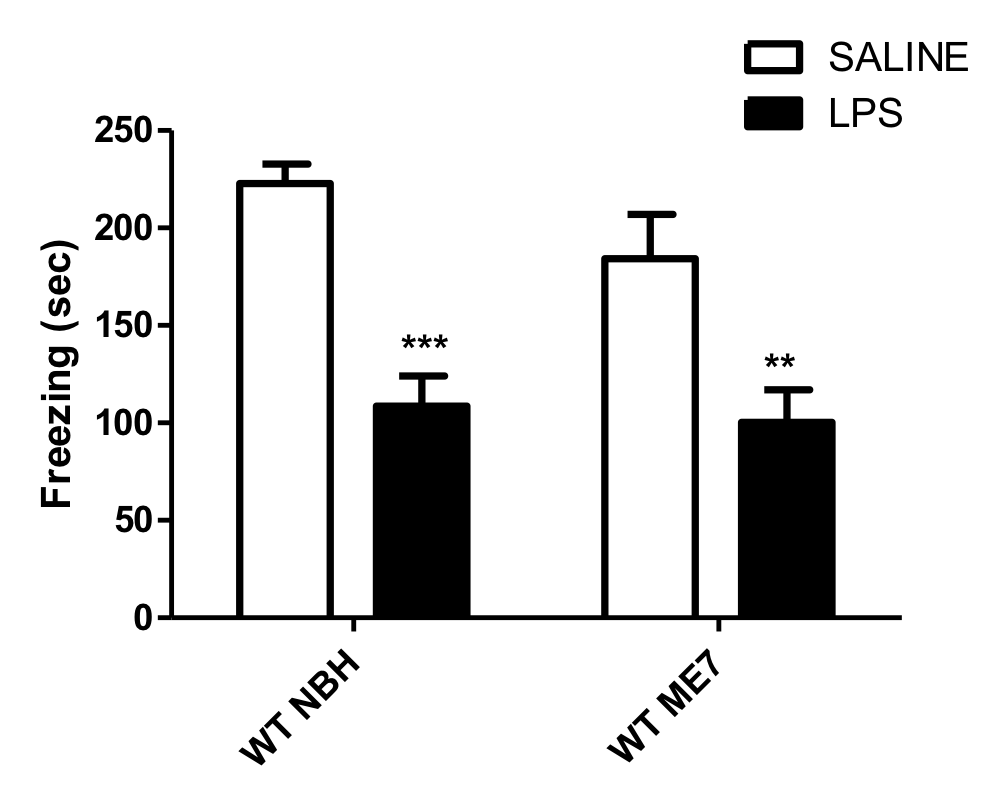


a) b)

**NBH ME7**

**Figure S1.** *Contextual fear conditioning in ME7 mice.*

Performance of WT NBH vs WT ME7 mice 16 wks post-inoculation in the contextual fear conditioning paradigm. The time spent freezing measured across 2 minutes (pre-shock) and 5 minutes, 48 hours later (post-shock), following foot shock at 0.4 mA for 2 seconds for WT NBH and WT ME7 mice (n=5) in CFC. Data are expressed as mean±SEM and were analysed by 2-way ANOVA with treatment and disease as factors. Bonferroni *post-hoc* tests after a significant effect of treatment in the ANOVA analysis showed that LPS treatment decreased freezing in both NBH and ME7 mice. Significant difference from controls (***p<0.001),(**p<0.01).

*Activation of cFOS in brain regions after LPS challenge in WT and IL-1RI^-/-^ mice*

It has previously been reported that sickness behaviour induced by peripheral challenge with LPS produces equivalent sickness behaviour patterns in wild-type and IL-1RI^-/-^ mice (Bluthe et al., 2000) and that these mice also show equivalent immune and acute phase responses to LPS (Glaccum et al., 1997). Given our observations on identical T-maze deficits after LPS in these mice (Figure 3a) we also sought to examine patterns of immediate early gene activation in brain centres responsive to peripheral LPS administration. These brain regions all showed highly similar patterns of activation in WT and IL-1RI^-/-^ mice as is shown in Table S1. The data confirm that largely equivalent responses to LPS occur in WT and IL-1RI^-/-^ animals despite the acknowledged role of IL-1 in sickness behavioural responses (Konsmann et al., 2008). These data indicate that there is redundancy in the cytokine dependence of sickness behaviour responses

**Supplementary Table 1**

cFOS activation of brain regions 3h after LPS (100 µg/kg i.p.) in normal and IL-1RI^-/-^ mice.

| **Group**  **Brain region** | WT + saline | WT + LPS | IL-1RI^-/-^ + saline | IL-1RI^-/-^ + LPS |
| --- | --- | --- | --- | --- |
| *Central Nucleus of Amygdala* | +/++ | +++/++++ | +/++ | ++++/+++ |
| *Sub-fornical organ* | + | +++ | + | +++ |
| *Lateral Parabrachial Nucleus* | + | +++ | + | +++ |
| *Area Postrema* | 0 | +++ | 0 | ++/+++ |
| *Nucleus Tractus Solitarus* | 0/+ | +++ | 0/+ | ++/+++ |
| *Ventrolateral medulla* | 0/+ | +/++ | 0/+ | +/++ |

*Inhibition of cytokine synthesis by Dexamethasone-21-phosphate*

Dexamethasone-21-phosphate significantly inhibits systemic synthesis of IL-1β (S1a), but does not have any significant impact on hippocampal transcription of IL-1β mRNA (S1b). Moreover, inhibition of the production of systemic cytokines using dexamethasone-21-phosphate does not affect LPS-induced apoptosis in brain coronal sections (S1c). This suggests that LPS successfully communicated an inflammatory signal to the brain and still induced brain cell apoptosis despite significantly lowered systemic cytokines.

**Supplementary Figure 2**

**Figure S2** *Impact of dexamethasone-21-phosphate on IL-1β and apoptosis.* a) Plasma IL-1β concentration, as measured by ELISA, in NBH and ME7 animals (18-19 weeks) at 4 hours post-LPS (500 µg/kg) ± pre-treatment with dexamethasone-21-phosphate (2 mg/kg); n=5 for NBH+LPS+dex and ME7+LPS+dex and n=4 for all other groups. b) TAQMAN quantitative PCR mRNA expression analysis of IL-1β; n=9 for ME7+saline, n=4 for NBH+dex and NBH+LPS and n=5 for all other groups. ** p<0.01, *** p<0.001 by Bonferroni post-hoc test after a significant main effect of treatment by one-way ANOVA. c) Number of TUNEL-positive cells per 10 µm coronal section (at the level of dorsal hippocampus); n=4 for NBH/ME7 + dex and n=5 for all other groups. All data have been presented as mean ± SEM except c where all values, and the mean, are shown. These data arise from the same preparation of dexamethasone-21-phosphate as the data in figure 3 (These data from Murray et al., Journal of Neuroinflammation (2011), used with permission from BioMed Central).


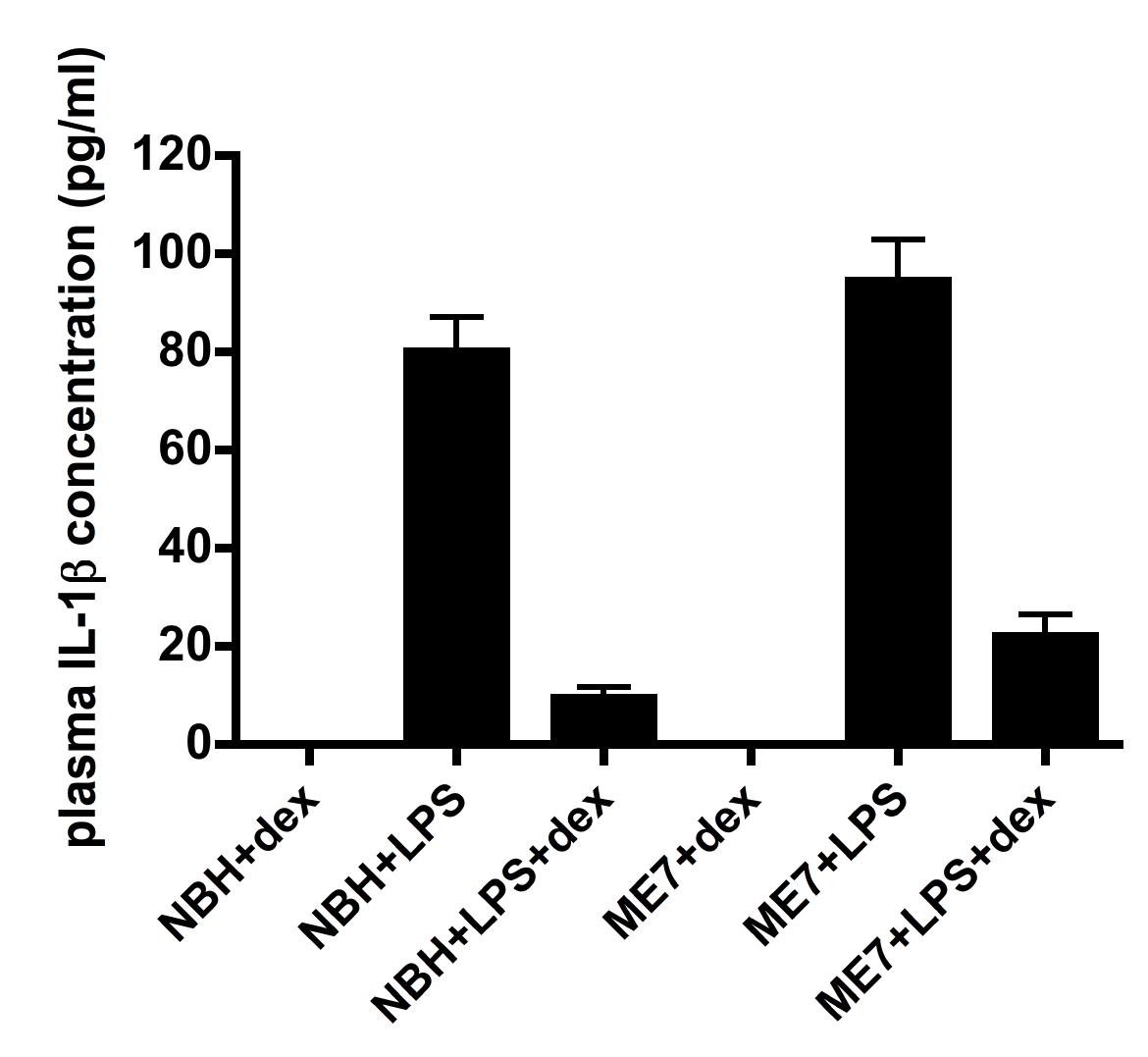

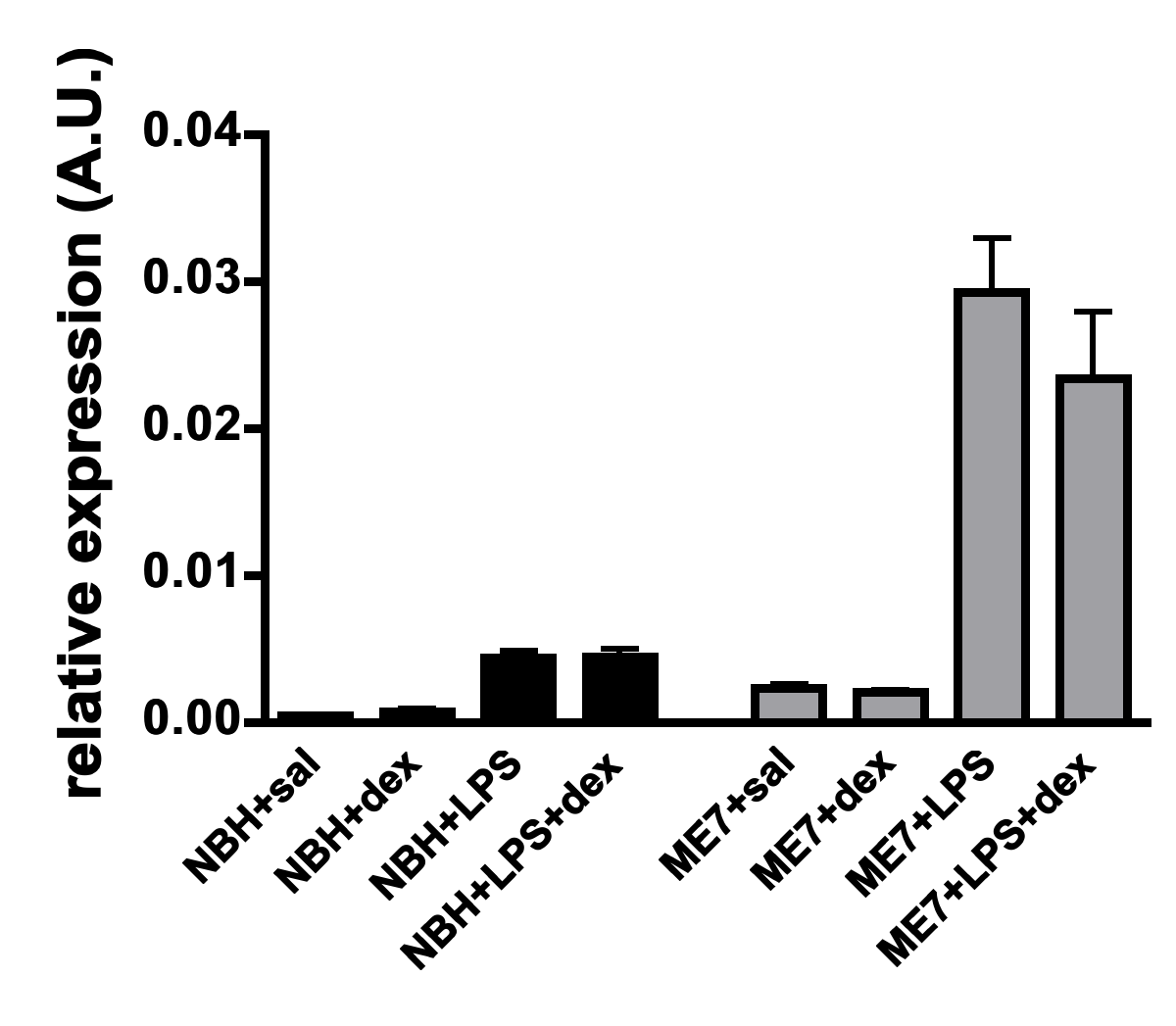

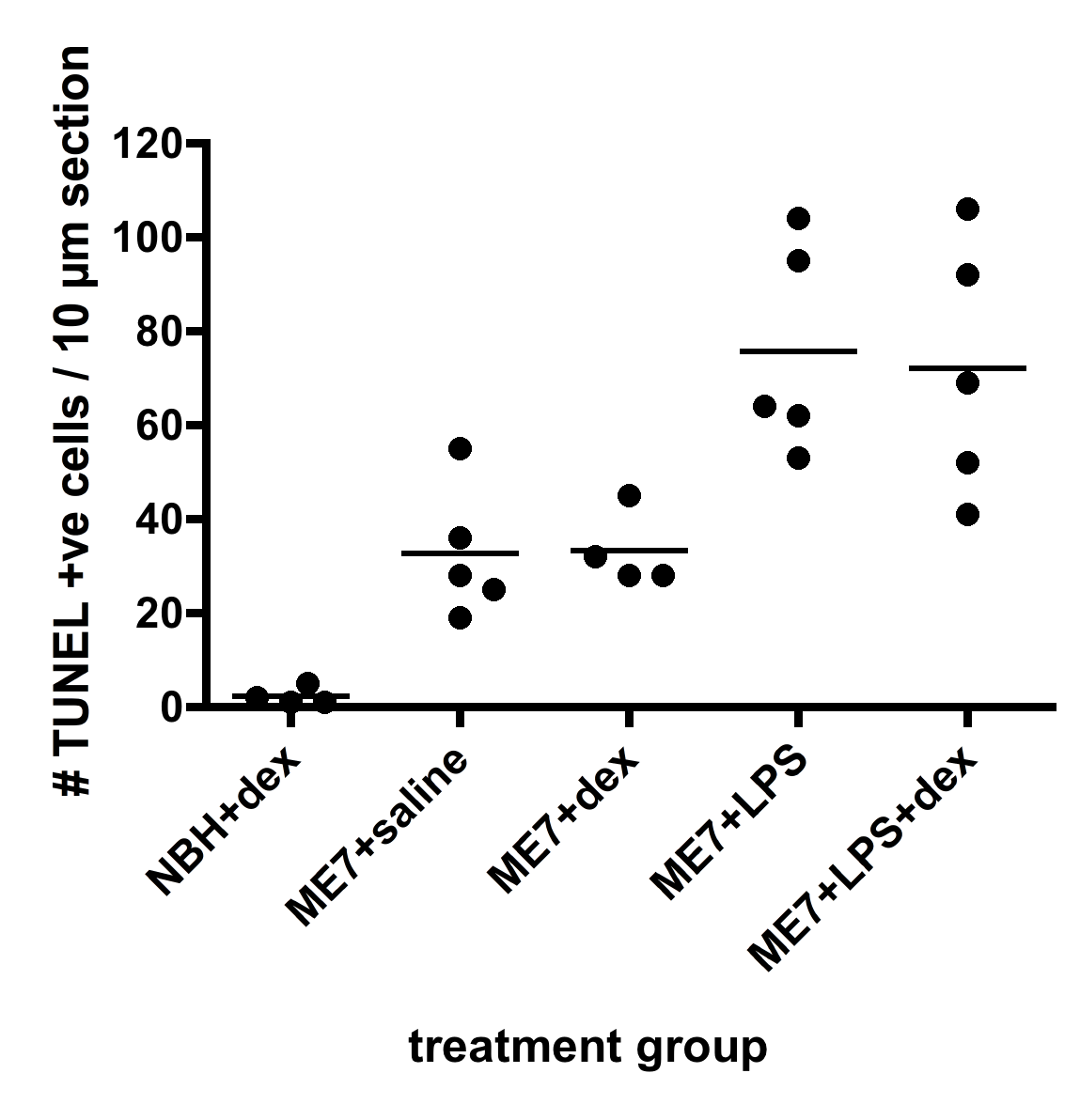


*Electrophysiology*

CA1 pyramidal neurons from ME7 animals recorded with a K^+^-based intracellular solution show a slightly depolarized membrane potential (Fig S3A). Using Cs-based intracellular solution, the resting membrane potential was similar to that of NBH animals (Fig S3A: -61 ± 3 mV, n = 3 cells), in agreement with previous findings (Johnston et al. (1998). That the membrane potential of ME7 neurons is normal with intracellular CsMeSO_4,_ which blocks potassium channels, indicates that the depolarised resting potential in recordings using the more physiological KMeSO_4_ intracellular solution is not attributable to impaired slice health or degeneration but due to a specific defect in a potassium conductance that regulates membrane potential. There were no differences in input resistance (240 ± 38 MΩ in NBH and 218 ± 30 MΩ in ME7, Fig. S2a) but cell capacitance was significantly reduced in ME7 cells (99 ± 7 pF in NBH and 72 ± 6 pF in ME7 cells, unpaired t-test, p = 0.006, Fig. S2c), consistent with reduced cell surface area and neurodegeneration.

**Supplementary Figure S3**

**
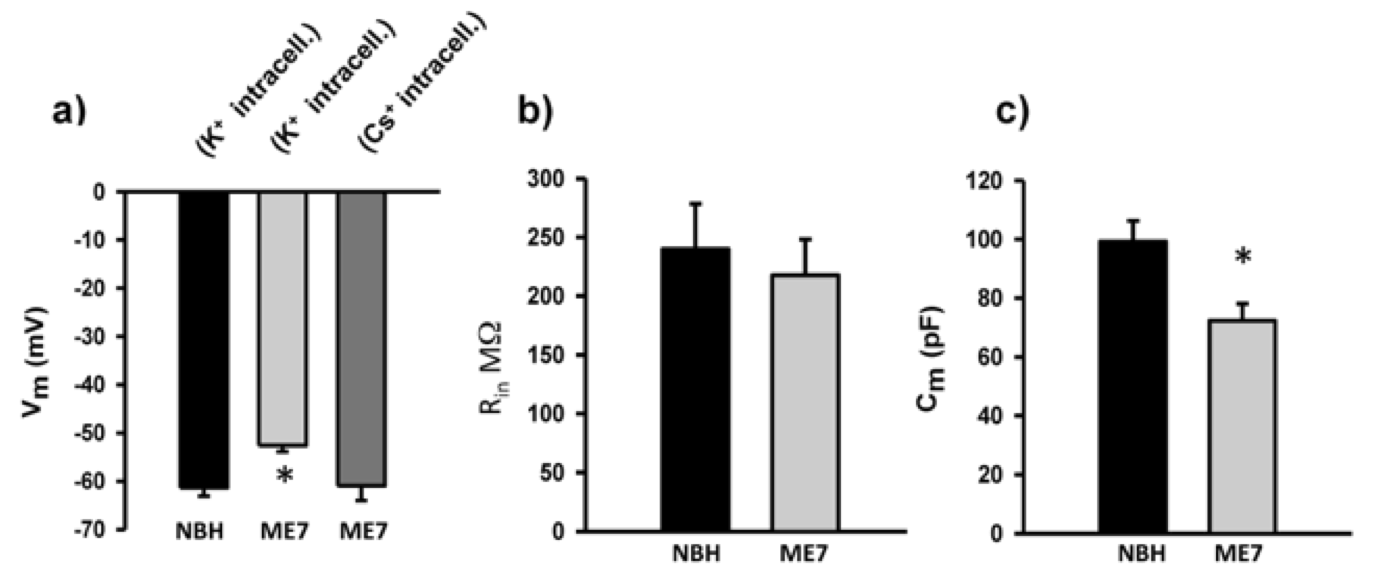
**

**Figure S3** *Depolarised resting membrane potential in neurons from ME7 animals.* (a) Resting membrane potential (V_m_) was significantly more depolarised in CA1 pyramidal cells from ME7 animals compared to NBH animals in whole-cell patch clamp recordings using KMeSO_4_ intracellular solution (n = 13 cells from 5 NBH animals and 24 cells from 8 ME7 animals, unpaired t-test, * p < 0.0001) but was normal in cells recorded with a Cs-based intracellular solution (n = 3 cells from 1 NBH animal). (b) Input resistance was not significantly different between cells from the two groups of animals. (c) Cell membrane capacitance was significantly reduced in cells from ME7 animals, n = 13 cells from 5 NBH animals and 24 cells from 8 ME7 animals, unpaired t-test, * p < 0.001. All data are mean ± SEM

*Non-synaptic depolarization induced by IL-1β but not TNF-α*

The depolarizing effect of IL-1β on resting membrane potential in ME7 neurons does not appear to be mediated by modulation of excitatory synaptic transmission. 1 ng/ml IL-1β had no effect on evoked excitatory postsynaptic currents (EPSC amplitude 105 ± 32 % of control amplitude, n = 4 cells from 2 ME7 animals; Fig S4). Similar non-synaptic depolarization was not induced using TNF-α at 20 ng/ml (Fig. S5).

**Supplementary Figure 4**

100 pA

10 ms

**Control**

**IL-1β (1 ng/ml)**

**Figure S4** *Depolarisation of ME7 neurons by IL-1β was not associated with modulation of excitatory postsynaptic currents (EPSCs).* Traces show averaged EPSCs that were unchanged by IL-1β at 1 ng/ml with EPSC amplitude measuring 105 ± 35 % of control (n = 4 cells from 2 ME7 animals).

**Supplementary Figure 5**

10 mV

5 ms

-61 mV

-64 mV

**Control**

**TNFα (20 ng/ml)**

**Control**

**TNFα**

**Figure S5** *TNFα did not affect membrane potential in neurons from IL-1R1^-/-^animals.* Traces illustrate current clamp recordings from CA1 pyramidal cells from IL-1R1^-/-^ animals in control conditions and in the presence of TNFα (20 ng/ml). The bar graph shows that mean membrane potential was not significantly different from control in TNFα (n = 5 cells from 2 animals, paired t-test, p = 0.88).

*Apoptosis in NBH animals 18 hours post-challenge with LPS.*

We examined whether LPS (750 µg/kg) induced significant apoptosis in NBH animals using TUNEL labelling and examined the IL-1RI-dependence of any observed effect. In this analysis, LPS had no significant effect on number of TUNEL-positive cells per coronal section (F_1,13_=0.01, p=0.9152) and strain (i.e. IL-1RI^-/-^) showed a trend towards decreasing apoptosis but this too was non-significant (F_1,13_=2.75, p= 0.1211). Likewise, there was no interaction between these factors (F_1,13_=0.25, p=0.6281). Therefore, LPS (750 µg/Kg) does not produce measureable new apoptosis in coronal sections, at the hippocampal level, in NBH animals (Fig. S6), in contrast to its effects in ME7 animals (Fig 5).

**Supplementary Figure 6**

**Figure S6.** *LPS-induced apoptosis in NBH animals*

NBH animals, on a wild-type or IL-1R1^-/-^ background, were challenged i.p. with LPS (750 µg/kg) or saline. Apoptotic cells were counted in 10 µm coronal sections. Data are shown as mean ± SEM with n=4 in all groups except LPS in IL-1RI^-/-^ (n=5). There were no statistically significant differences to annotate. Two way ANOVA analysis of strain and treatment (effects and interactions detailed in main supplementary text).

*Exaggerated hypothermic response in ME7 animals treated with LPS.*

Based on the hypothesis that microglial priming leads to exaggerated IL-1 synthesis in the brain of animals with existing neurodegeneration and the data in Fig. 4 demonstrating heightened sensitivity of ME7 neurons to IL-1 stimulation, we hypothesised that ME7 animals would show exaggerated hypothermic responses to LPS and that this exaggeration would be mediated by IL-1RI. Animals were assessed for core-body temperature directly before euthanisation at 18 hours post-LPS. LPS-treated NBH animals still showed significant hypothermia at this time, but this effect was very much exaggerated in ME7+LPS animals (Fig. S7; p<0.01). In IL-1R1^-/-^ ME7+LPS animals, this exaggerated effect was very significantly mitigated (p<0.01; all analyses by selected Bonferroni pairwise comparisons after a significant interaction of disease, strain and treatment by 3 way ANOVA: F_1,33_=7.97, p=0.008). Therefore, the exaggerated sickness induced by LPS in ME7 animals is to a substantial extent mediated via IL-1R1.

**Supplementary Figure 7**


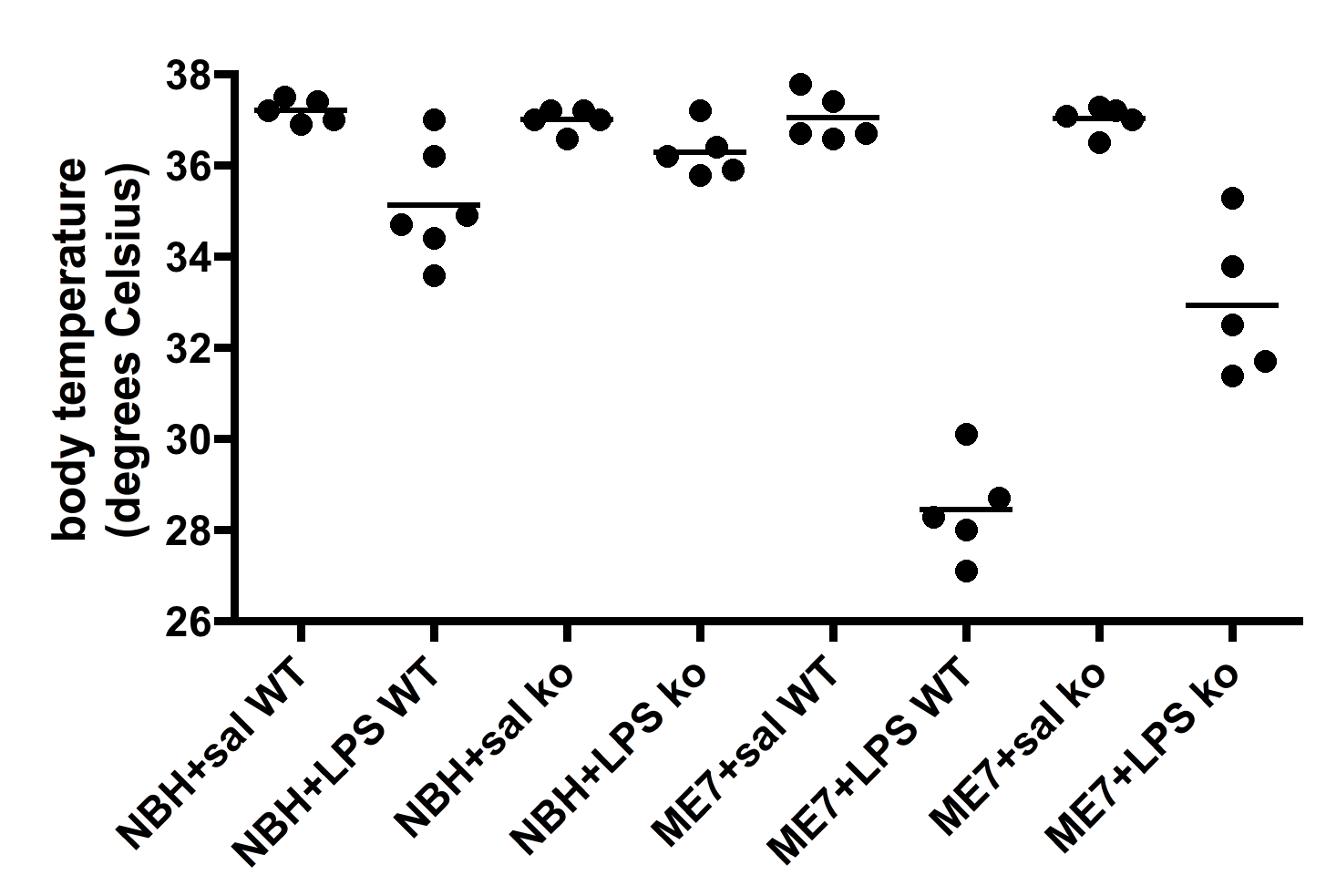


***

**Figure S7.** *LPS-induced hypothermia is exaggerated and IL-1RI-dependent in ME7 animals.* WT and IL-1RI^-/-^ (ko) animals were assessed (immediately before euthanisation at 18 hours) for core-body temperature. Data are shown as scatter dot plots with the mean displayed. *** denotes ME7+LPS was significantly different both to NBH+LPS and to ME7 IL-1R1^-/-^+LPS (p<0.001) by Bonferroni post-hoc test after significant 3 way ANOVA).

**Supplementary Reference**

Johnston, AR, Fraser, JR, Jeffrey, M, and MacLeod, N, *Alterations in potassium currents may trigger neurodegeneration in murine scrapie.* Exp Neurol, 1998. **151**(2): p. 326-33.
